# Viscoelastic Effects on the Response of Electroelastic Materials

**DOI:** 10.3390/polym13132198

**Published:** 2021-07-01

**Authors:** Ricardo Diaz-Calleja, Damián Ginestar, Vícente Compañ Moreno, Pedro Llovera-Segovia, Clara Burgos-Simón, Juan Carlos Cortés, Alfredo Quijano, Joaquín Díaz-Boils

**Affiliations:** 1 Instituto de Tecnología Eléctrica, Universitat Politècnica de València, 46022 Valencia, Spain; pedro.llovera@ite.es (P.L.-S.); alfredo.quijano@ite.es (A.Q.); 2 Instituto Universitario de Matemática Multidisciplinar, Universitat Politècnica de València, 46022 Valencia, Spain; dginesta@mat.upv.es (D.G.); c.burgos.simon@gmail.com (C.B.-S.); jccortes@mat.upv.es (J.C.C.); 3 Departamento de Termodinámica Aplicada, Universitat Politècnica de València, 46022 Valencia, Spain; vicommo@ter.upv.es; 4 Instituto Tecnológico de la Energía-Redit, 46980 València, Spain; 5 Facultad de Ingeniería, Universidad Internacional de la Rioja, 26002 La Rioja, Spain; joaquin.diaz@unir.net

**Keywords:** viscoelasticity, electroelastic materials, fractional derivatives, bifurcations

## Abstract

Electroelastic materials, as for example, 3M VHB 4910, are attracting attention as actuators or generators in some developments and applications. This is due to their capacity of being deformed when submitted to an electric field. Some models of their actuation are available, but recently, viscoelastic models have been proposed to give an account of the dissipative behaviour of these materials. Their response to an external mechanical or electrical force field implies a relaxation process towards a new state of thermodynamic equilibrium, which can be described by a relaxation time. However, it is well known that viscoelastic and dielectric materials, as for example, polymers, exhibit a distribution of relaxation times instead of a single relaxation time. In the present approach, a continuous distribution of relaxation times is proposed via the introduction of fractional derivatives of the stress and strain, which gives a better account of the material behaviour. The application of fractional derivatives is described and a comparison with former results is made. Then, a double generalisation is carried out: the first one is referred to the viscoelastic or dielectric models and is addressed to obtain a nonsymmetric spectrum of relaxation times, and the second one is the adoption of the more realistic Mooney–Rivlin equation for the stress–strain relationship of the elastomeric material. A modified Mooney–Rivlin model for the free energy density of a hyperelastic material, VHB 4910 has been used based on experimental results of previous authors. This last proposal ensures the appearance of the bifurcation phenomena which is analysed for equibiaxial dead loads; time-dependent bifurcation phenomena are predicted by the extended Mooney–Rivlin equations.

## 1. Introduction

Recently, viscoelastic models have been proposed to give an account of the dissipative behaviour of the electroelastic materials [1,2,3,4,5,6,7,8,9,10,11,12]. This is justified because the range of temperatures at which these materials are being currently used is close to the glass transition temperature. Indeed, above the glass transition temperature, the materials in a rubber-like state still exhibit some rest of the viscoelastic behaviour. As a consequence, in these materials, the response to an external mechanical or electrical force field implies relaxation processes towards a new state of thermodynamic equilibrium. The characteristic time required for these relaxation processes is called the relaxation time. The literature on the subject concerning mechanical and dielectric behaviour is extensive (see, for example, [13,14,15,16,17,18]). The more typical mechanical experiments are called creep or stress relaxation according to the input type (stressing or stretching). The same occurs in the case of the dielectric relaxation or retardation experiments.

In order to represent the most elementary relaxation or retardation processes, different types of rheological or dielectric models have been proposed. These models consist of a short number of springs and dashpots for the mechanical case or capacitors and resistances, for the dielectric counterpart. These models are appealing in the sense that they allow easy visualisation of the corresponding mechanical or electrical relaxation processes. However, they are only a crude representation of the actual behaviour of the system under study. In fact, most of the proposed models are related to systems governed by a single relaxation time. This problem may be solved [13,16] by adding, in series or parallel, depending on the problem, a finite set of these viscoelastic or dielectric models. This is equivalent to assuming a discrete distribution of relaxation times. However, instead of a discrete distribution of relaxation times, it seems more realistic to consider a continuous distribution or spectra of these times. In fact, it is well known that, in practice, viscoelastic and dielectric materials, as is the case of polymers, exhibit a distribution of relaxation times instead of a discrete set of relaxation times [13,15].

The molecular origin for this distribution may be due to the different microscopic moieties involved in the process, or else to a distribution of the shape and size of the defects associated with the neighbourhood surrounding the molecular moieties. The macroscopic result is, in any case, a broad relaxation spectrum of these relaxation times. For this reason, in the present approach, a continuous distribution is considered.

On the other hand, in the theoretical background, a substantial modification of the classical model equations for the purely electroelastic materials is necessary in order to consider the viscoelastic and dielectric relaxation effects both of which are intrinsically dissipative.. In this sense, an approach based on classical irreversible thermodynamics has been proposed by Suo et al. [4]. Indeed, these authors assume that the response of an elastomer to mechanical and electrical force fields is delayed in time due to the relaxation mechanisms associated with the motion of the structural moieties. The basic idea is to introduce into the free energy density convenient additional parameters, taking the form of internal variables, as in the classical irreversible thermodynamics (CIT), describing the different degrees of freedom associated with the dissipative relaxation processes. In the same way, the analysis of the electromechanical instability requires taking into account, together with the kinematic variables, the new internal ones just introduced. The basic lines of this approach have been followed by other authors [6,8]. The theory has been tested by assuming a rheological model governed by a single relaxation time in the framework of a neo-Hookean model. In particular, the pertinent viscoelastic data for the VHB 4910 are obtained from earlier papers by Wissler and Mazza [19] and Planté and Dubowsky [20]. In the approach used by Suo et al. [4], only mechanical dissipation has been taken into account. This is justified on the basis that the mechanical relaxation times are several orders of magnitude larger than the dielectric counterpart retardation times and, for this reason, the viscoelastic relaxation process takes place at several orders of magnitude larger than the dielectric ones. Consequently, the highest dielectric relaxation in the time domain, which is the space charge relaxation, has been completely relaxed. In the present approach, the viscoelastic data are obtained, as explained later, from the paper by Wang et al. [7], and incompressible materials are assumed. In any case, it should be mentioned that the dielectric relaxation processes can be addressed in the same way as the counterpart viscoelastic ones.

Recently, Ghosh and Lopez-Pamies [9] have proposed a two-potential framework in order to model the viscoelastic–dielectric elastomers. In this case, both the viscoelastic and dielectric relaxations are accounted for. The functional form of these two potentials allows electromechanical coupling and creep and relaxation processes after, respectively, applying and removing the mechanical and electrical inputs. The first potential is used for free energy and the second for dissipation. In this second case, the reduced dissipation inequality, for the case of isothermal processes, imposes additional constraints on the dissipation potential. The constitutive model used in [9] had been previously proposed by Lopez-Pamies [21], and it is expressed in terms of only the first invariant of the right Cauchy–Green deformation tensor. As in [4], a single relaxation time has been assumed for the viscoelastic as well as for the dielectric relaxation.

On this background, attention is paid to enlarge the viscoelastic and dielectric effects in electroelastic materials to consider the case of a distribution of relaxation times. For this purpose, the simplified approach used in Ref. [4] is followed in the present paper. Our formal strategy is to introduce the classical linear differential equations governing the relaxational behaviour of nonlinear viscous terms instead of the classical Newtonian ones. These nonlinear terms take the formal structure of fractional derivatives [22] which physically correspond to constant phase elements (CPEs), as defined in Appendix A in the structure of the equations governing the relaxational (retardational) processes. It should be noted that some progress in this direction has been made by Xiao et al. [23]. The constant phase elements induce the appearance of distributions (spectra) of relaxation (retardation) times. Moreover, since these distributions do not exhibit in practice symmetrical shape, the dynamic equations should be modified accordingly. Specifically, these modifications result in the introduction of two constant phase elements, respectively, for the low- and high-frequency sides of the spectrum.

Accordingly, the plan of the paper is as follows: after the introductory remarks in this section, in Section 2, the background of the paper is developed. In Section 3, the formalism of the fractional derivatives is introduced in the viscoelastic or dielectric models in order to give an account of a distribution of relaxation times. Moreover, the formal treatment in terms of fractional derivatives is developed. In Section 4, a double generalisation is made. The first one refers to viscoelastic or dielectric models and is addressed in order to obtain a nonsymmetric spectrum of relaxation times. The second is the adoption of the more realistic Mooney–Rivlin equation, instead of the classical neo-Hookean model, for the stress–strain relationship of the elastomeric material. Consequently, the theory developed in [4] is modified accordingly for the cases of compliant and floating electrodes. It should be noted that the adoption of the Mooney–Rivlin model equation ensures the appearance of the bifurcation phenomena. In Section 5, a practical example including a discussion on the dynamic mechanical measurements is analysed. In particular, the acrylic elastomer VHB 4910 from 3M is considered as usual. Section 6 is devoted to the numerical strategy for the calculations. In particular, the case where the system is subjected to an external electric force field but not to mechanical loadings is studied. A comparison between the results obtained by considering neo-Hookean and Mooney–Rivlin materials governed by a single relaxation time and the present results for the distribution of relaxation times is also made. Section 7 is devoted to analysing the effect of viscoelastic relaxation on the bifurcation processes appearing in the material under study. Section 8 and Section 9 are, respectively, concerned with a discussion of the results and the conclusions of the present research.

## 2. Theoretical Background

Elastomers show a nonlinear hyperelastic behaviour superimposed with dissipative viscoelastic and dielectric processes. The presence of these dissipative processes is due to the fact that the temperatures at which these materials are in use remain, in many cases, close to the glass transition temperature at which the viscoelastic and dielectric effects are still present. For example, let us consider the case of the VHB 4910, one of the materials that have merited preferential attention by part of the researchers due to its applicability. According to our experimental data [24], the glass transition temperature of the VHB 4910 measured by differential scanning calorimetry (DSC) at 20 °C/min is close to –37 °C. Although the tensile stress–strain analysis is carried out at 25 °C, it is still possible to detect experimentally some viscoelastic effects in the material behaviour. In any case, dynamic mechanical data should be the most convenient ones to analyse the present problem, as discussed later.

It is well known that the glass transition is detected in dynamic mechanical experiments by a drastic diminution in the viscoelastic modulus of pure amorphous polymers of about three decades in the value of the elastic modulus. However, in the case of the VHB 4910, only a diminution of about half a decade is observed. This fact can be due to the chemical structure of the material, or to the residual character of the viscoelastic effects. Moreover, an additional experimental problem appears due to the fact that at the temperatures above the glass transition the material is too soft. This fact frequently precludes obtaining reliable and reproducible dynamic results. In these conditions, the dynamic mechanical data should be derived from alternative techniques. In practice, many of the dynamic results are obtained indirectly from uniaxial extension experiments giving the extensional (Young) modulus as a function of the time or, alternatively, from the shear modulus. These facts are probably in the origin of the dispersion of the dynamic viscoelastic data at hand, as will be discussed later.

**Figure 1:**
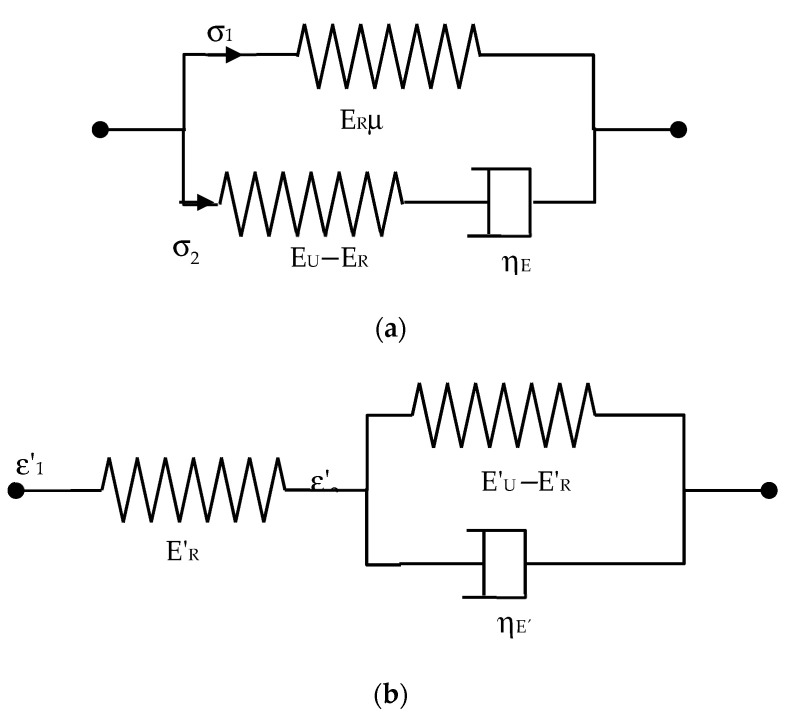
Standard solid model representing electromechanical behaviour: (**a**) spring in parallel with a Maxwell element, (useful for strain inputs); (**b**) spring in series with a Kelvin-Voigt element (useful for stress inputs).

On the other hand, the classical nonlinear elastic models are time independent and, consequently, do not give an account of the viscoelastic behaviour which is frequency (time) dependent. As mentioned above, Suo et al. [4] have recently proposed an approach to give an account of the dissipative viscoelastic behaviour of the dielectric elastomers based on the CIT. On this basis, they have constructed a theory that, as a particular case, includes the annihilation of the classical hessian determinant to determine the stability or instability conditions. The aforementioned theory requires new ingredients to give an account of the irreversible processes suffered by the material during the relaxation processes. Then, a set of internal variables are included as a part of the Helmholtz free energy density. Subsequently, a differential equation to represent the kinetics of the actual irreversible processes appears. This differential equation needs to be solved in conjunction with the constitutive stress–strain relations derived from the free energy density. Suo et al. rightly state that ‘there is considerable flexibility in choosing kinetic models to fulfil the thermodynamic inequality’, which, in fact, is derived as a consequence of the second law of thermodynamics. In any case, the choice should be experimentally consistent. On this basis, an order one kinetic has been chosen to illustrate the general approach. A first-order kinetic, which is represented by a first-order differential equation, is equivalent to considering a single relaxation time associated with the relaxation process. This relaxation time is related to the dashpot appearing in the classical solid standard model (Zener model). It should be stressed that two types of standard solid models are possible. One is a spring in parallel with a Maxwell element, that is, a spring with a dashpot in series (Figure 1a), and it is used in this paper as the basis of the more complex models. The second one is a spring in series with a Kelvin–Voigt element, which is a spring in parallel with a dashpot (Figure 1b), and it has been used by other authors [2,7]. The first model is related to the case of a strain input (stress relaxation experiment), whereas the second one corresponds to the case in which a stress input is used (creep experiment). The parameters of both models are obviously related [15], and similar models have been proposed for the case of dielectric relaxation processes [18].

In this respect, the simplest dielectric model corresponds to the results of the classical Debye theory of the dielectric relaxation based on the rotational Brownian motion [25], which considers a single relaxation time. In fact, the Debye equivalent electric circuit includes two capacitors representing the passive elements for the storage of the electrical energy and a resistor to give an account of the dissipative process. The mechanical equivalent circuit in the electromechanical analogy is, of course, the above-mentioned Zener model consisting of two springs and a dashpot. However, the single relaxation time model initially proposed by Debye requires modification in order to fit the experimental results of the materials under study because of the actual presence of a distribution of relaxation times. A first alternative model was proposed by Cole and Cole [26] after studying the dielectric properties of some alcohols, showing non-Debye behaviour. This model implies the substitution of the resistor in the electric circuit by a CPE, which is an empirical impedance function (see Appendix A) and has a parabolic form in the time domain as can be found in the literature [16,18]. It is noticeable that the introduction of a CPE induces the appearance of a distribution of relaxation times. However, in the case of polymeric materials, the experimental data and the corresponding relaxation time spectra show a nonsymmetric shape. As a consequence, Havriliak and Negami (HN) [27] have modified the Cole–Cole model (CC) [26] to represent accurately the actual dielectric relaxation data of most of the polymers. The HN model predicts in fact a nonsymmetric relaxation time spectrum in agreement with the nonsymmetric shape of the experimental data and has been extensively used in practice. As an alternative to the HN equation, a biparabolic model has been proposed by Huet [28,29], which includes two CPEs. Each one of these CPE accounts for, respectively, the low- and high-frequency sides of the spectra. It should be noted that the parameters of the HN equation are closely related to those of the biparabolic equation [18] (p. 205). The equivalent mechanical model is easy to obtain, taking into account that the permittivity is, in the electromechanical analogy, mechanical compliance and assuming that the corresponding mechanical counterpart is an electric modulus. It should be noted that in mechanical terms the adoption of a CPE is equivalent to assume, instead of a simple Newtonian behaviour, a non-Newtonian one for the mechanical dissipative element. On this basis, a modification of the kinetic model used in [4] is pertinent.

## 3. Formal Analysis

The starting point to the formal analysis of the viscoelastic behaviour is the standard solid model proposed in [4] (Figure 1a), which is formed by a spring in parallel with a Maxwell element, that is, a spring in series with a dashpot. However, due to the fact that the viscoelastic primary data are obtained from uniaxial tensile modulus, the parameters of the standard solid model refer to the relaxed and unrelaxed tensile moduli and the corresponding tensile viscosity (respectively, E_R_, E_U_, and η_E_).

The differential equation governing this model is given by

(1) $$\left(E_{R}+\tau_{E}E_{U}\frac{d}{\mathrm{dt}}\right)\epsilon =\left(1+\tau_{E}\frac{d}{\mathrm{dt}}\right)\sigma$$

where σ and ϵ are, respectively, the stress and the strain, $\tau_{E}=\eta_{E}/\left(E_{U}-E_{R}\right)$ is the characteristic relaxation time, $\eta_{E}$ ($\eta_{E}>0$) is the viscosity coefficient associated with the dashpot, and $E_{R},{\;E}_{U}$ are, respectively, the relaxed and unrelaxed tensile modulus. In forming this differential equation (see Ref. [15]), the following relation between the stress in the Maxwell element in parallel and the strain in the dashpot is used:

(2) $$\sigma_{2}=\eta_{E}\dot{\epsilon }$$

where a superscript dot indicates time derivative. Equation (2) corresponds to the classical Newtonian behaviour in the dashpot.

The stress response of the model given by Equation (1) in the time domain to a strain input given by $\epsilon =\epsilon_{0}H\left(t\right)$, where H(t) is the unit Heaviside function (step function), is given by

(3) $$\sigma \left(t\right)=\epsilon_{0}\left[E_{R}+\left(E_{U}-E_{R}\right)\exp\left(-t/\tau_{E}\right)\right]$$

This seems to be the type of response depicted in Figure 2b of [4].

Then, the corresponding relaxation modulus in the time domain is given by

(4) $$\;E\left(t\right)=E_{R}+\left(E_{U}-E_{R}\right)\exp\left(-t/\tau_{E}\right)$$

and the dynamic modulus in the frequency domain is obtained from the Laplace transform of both sides of (4) and is defined by

(5) $$E^{*}=\mathrm{sE}\left(s\right)=E_{R}+\frac{\left(E_{U}-E_{R}\right){s\tau }_{E}}{1+{s\tau }_{E}}=E_{R}+\frac{\left(E_{U}-E_{R}\right)}{1+{\left({s\tau }_{E}\right)}^{-1}}$$

where s=jω, $E\left(s\right)=\sigma \left(s\right)/\epsilon_{0}$, and $E^{*}=\sigma \left(s\right)/\epsilon \left(s\right)$ is the defined dynamic modulus.

This is the classical single relaxation time dynamic response in the frequency domain corresponding to the standard solid model.

**Figure 2:**
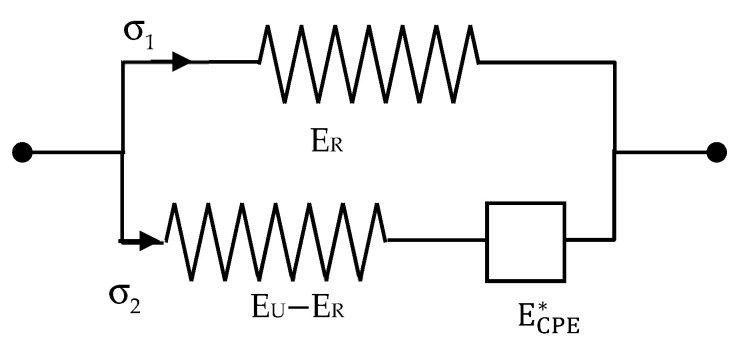
Modified solid standard model representing electromechanical behaviour with a mechanical constant phase element (CPE).

However, in order to give an account of the observed distribution of relaxation times experimentally observed, there are several possibilities, but in the present case, a slightly more sophisticated model is considered, as the one shown in Figure 2, where a mechanical CPE instead of a dashpot is used. The empirical mechanical impedance of this CPE may be expressed in terms of the parameters shown in Figure 2 as

(6) $$E_{\mathrm{CPE}}^{*}=\left(E_{U}-E_{R}\right){\left({s\tau }_{E}\right)}^{\alpha }$$

where α∈(0,1).

Taking into account Equation (6), the total mechanical impedance of the scheme, represented in Figure 2, may be found as the addition of the impedance of the upper branch of the scheme in that figure and the inverse of the addition of the inverses of the impedance of the two elements of the lower branch of the same figure.

(7) $$E^{*}=E_{R}+\frac{\left(E_{U}-E_{R}\right)E_{\mathrm{CPE}}^{*}}{\left(E_{U}-E_{R}\right)+E_{\mathrm{CPE}}^{*}}=E_{R}+\frac{\left(E_{U}-E_{R}\right){\left({s\tau }_{E}\right)}^{\alpha }}{1+{\left({s\tau }_{E}\right)}^{\alpha }}=E_{R}+\frac{\left(E_{U}-E_{R}\right)}{1+{\left({s\tau }_{E}\right)}^{-\alpha }}$$

Since s represents in the time domain the first-order derivative with respect to the time, $s^{\alpha }$ can be interpreted in the present context as a fractional derivative of order α. The introduction of the constant α is enough to create a distribution of relaxation times corresponding to the viscoelastic model given by Equation (7).

It can be shown that this result should be obtained by assuming a new relation between the stress and the rate of strain in the dashpot as follows:

(8) $$\sigma_{2}=\eta_{E}\frac{d^{\alpha }\varepsilon_{2}}{{\mathrm{dt}}^{\alpha }}$$

where $\sigma_{2}$ and $\varepsilon_{2}$ are, respectively, the stress in the bottom branch of the scheme shown in Figure 2, and the strain in the CPE of this Figure, and $\eta_{E}$ is a pseudo-viscosity.

Equation (8) may be considered as corresponding to a ‘fractional dashpot’ where α=0, 1 refer, respectively, to the ideal plasticity and the Newtonian liquid. In our case, being 0<α<1 the obtained result in the time domain corresponds to the situation of nonexponential stress relaxation, as observed in the actual experiments.

Of course, more complex models different from that given by Equation (8) should be assumed as it will be shown later. Then, taking into account Equation (8), the differential Equation (1) is modified, and thereafter, the pertinent algebra can be reformulated according to (see Appendix B)

(9) $$\left(E_{R}+E_{U}\tau_{E}{}^{\alpha }\frac{d^{\alpha }}{{\mathrm{dt}}^{\alpha }}\right)\epsilon =\left(1+\tau_{E}{}^{\alpha }\frac{d^{\alpha }}{{\mathrm{dt}}^{\alpha }}\right)\sigma$$

where $\tau_{E}{}^{\alpha }=\frac{\eta_{E}}{E_{U}-E_{R}}$, being $\eta_{E}$ the pseudo-viscosity, and $\frac{d^{\alpha }}{{\mathrm{dt}}^{\alpha }}f\left(t\right)$ denotes the fractional derivative of order 0<α≤1 of a function f(t).

It is a well-known fact that there are several kinds of fractional derivatives [22]. In this work, we have chosen the Caputo derivative because its properties make it possible to consider initial conditions in terms of the value of the function f(t) as is required in our context. The intuitive meaning of Caputo derivative lies in the concept of fractional integral, termed Liouville fractional integral, which is defined by

(10) $$(J_{0}^{\alpha }f)\left(t\right)=\frac{1}{\Gamma \left(\alpha \right)}\int_{0}^{t}{\left(t-s\right)}^{\alpha -1}f\left(s\right)\mathrm{ds}$$

where Γ(α) is the Euler’s Gamma function. Then, the Caputo derivative is defined by

(11) $$\;\frac{d^{\alpha }}{{\mathrm{dt}}^{\alpha }}f\left(t\right)=(J_{0}^{n-\alpha }f^{\left(n\right)})\left(t\right)\;$$

where n=−[−α], (being [−α], the greatest integer less than or equal to (−α), and $f^{\left(n\right)}\left(t\right)$ denotes de n-th ordinary classical derivative of f(t). Intuitively, in order to obtain the Caputo fractional derivative of f(t) of order α, first, it is necessary to differentiate n times the function f(t) and then to integrate the exceeding part. This latter term, consisting of calculating an integral (thus involving the evaluation of the function f(t) over its integration domain), allows the interpretation of the Caputo derivative as a ‘memory’ operator since it takes into account the physical ‘history’ described by the function f(t). In numerous physical contexts (see, for example, Refs. [30,31]), the Caputo derivative has demonstrated to successfully capture the ‘past history’ of the corresponding phenomena under study described via the function f(t). Furthermore, it is easy to check, by means of the application of integration by parts the formula to $(J_{0}^{\alpha }f)\left(t\right)$, that the Caputo derivative becomes the ordinary derivative when α is a positive integer, thus retaining the physical meaning of the classical derivative.

In our case, we consider that 0<α≤1; thus, the Caputo derivative of this specific order is given by

(12) $$\frac{d^{\alpha }}{{\mathrm{dt}}^{\alpha }}f\left(t\right)=\frac{1}{\Gamma \left(\alpha \right)}\;\int_{0}^{t}\frac{f^{\prime }\left(s\right)}{{\left(t-s\right)}^{1-\alpha }}\mathrm{ds},\;0<\alpha \leq 1,\;t\;>0.$$

Differential models in viscoelasticity including fractional derivatives were suggested by several authors [32,33,34,35,36,37] and used in connection with the viscoelastic or dielectric time-temperature superposition [38].

**Figure 3:**
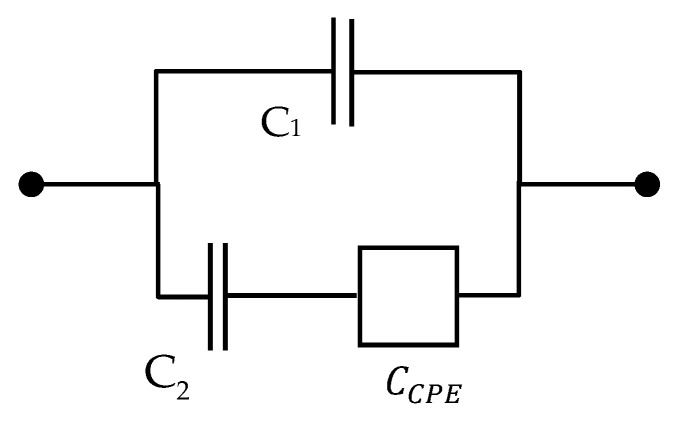
Dielectric counterpart of the mechanical model with a CPE.

It is noteworthy that the dielectric counterpart of the mechanical model (Appendix A) consists of a capacitor with capacitance $C_{1}$ in parallel with a series coupling of a capacitor with capacitance $C_{2}$ and a CPE, as represented in Figure 3.

## 4. Generalisation for Non-Symmetric Relaxation Spectrum

**Figure 4:**
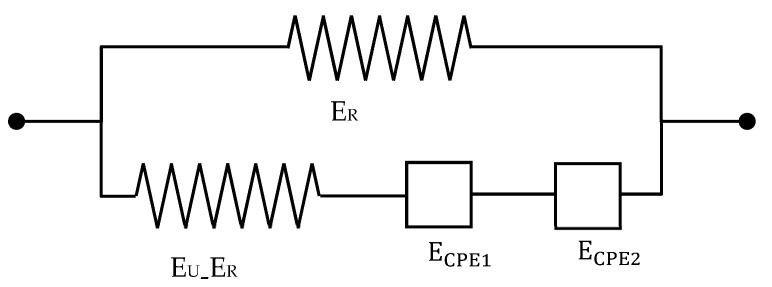
Modified solid standard model representing a nonsymmetric distribution of relaxation times.

As mentioned above, Equations (7) and (A5) predict a symmetric distribution of relaxation times [18], which is unlikely for polymeric materials. For this reason, a biparabolic model with two constant phase elements is proposed to represent the dynamic shear viscoelastic data [18]. The corresponding mechanical model is shown in Figure 4.

In a similar way to how is conducted in Equation (6), one can write

(13) $$E_{1\mathrm{CPE}}^{*}=\left(E_{U}-E_{R}\right){\left({s\tau }_{E}\right)}^{\alpha },\;E_{2\mathrm{CPE}}^{*}=\left(E_{U}-E_{R}\right)\delta^{-1}{\left({s\tau }_{E}\right)}^{\beta }$$

for, respectively, the low- and high-frequency side of the spectrum. This explains why only two continuous phase elements at least are necessary.

Thus, after the pertinent calculations similar to those of Appendix B, the dynamic tensile modulus can be expressed as

(14) $$E^{*}=\mathrm{sE}\left(s\right)=E_{R}+\frac{\left(E_{U}-E_{R}\right)}{1+{\left({s\tau }_{1}\right)}^{-\alpha }+\gamma {\left({s\tau }_{1}\right)}^{-\beta }}=E_{R}+\frac{\left(E_{U}-E_{R}\right)}{1+{\left({s\tau }_{1}\right)}^{-\alpha }+{\left({s\tau }_{2}\right)}^{-\beta }}$$

where 0<β<α<1, $\tau_{1}=\tau ,{\;\tau }_{2}={\tau \gamma }^{-1/\beta }$, and γ>0 is a parameter associated with the nonsymmetric shape of the spectrum [39].

At this point, it is important to note that the viscoelastic parameters appearing in Equation (14) refer to the dynamic tensile modulus, whereas the elastic parameters that appear in the free energy density correspond to the shear modulus. In general, the relationship between the tensile and the shear viscoelastic moduli is given by

(15) $$E^{*}=2\mu^{*}\left(1+\nu^{*}\right)$$

where $\nu^{*}$ is the viscoelastic Poisson ratio. For incompressible materials, the Poisson ratio is commonly taken as $\nu^{*}=0.5$ due to the incompressibility assumption. For this reason, in those that follow and in order to relate the dynamic viscoelastic tensile modulus to the dynamic shear modulus, the following equation is taken:

(16) $$E^{*}=3\mu^{*}$$

In these conditions, in those that follow, a similar expression to that of Equation (14) is taken for the shear modulus, that is,

(17) $$\mu^{*}=\mu_{R}+\frac{\left(\mu_{U}-\mu_{R}\right)}{1+{\left({s\tau }_{1}\right)}^{-\alpha }+{\left({s\tau }_{2}\right)}^{-\beta }}$$

The use of the shear modulus μ is justified because from the Hooke’s law and the relation between the stretching λ and the deformation ϵ for relatively small deformations, the classical result $\mu ={\mathrm{Nk}}_{B}T$ is obtained, where N is the number of chains of the network, $k_{B}$ is the Boltzmann constant and T the absolute temperature. As will be seen later (see Equations (31) and (32)), the shear modulus of the material is closely related to the parameters $\mu_{\mathrm{Ui}}$ and $\mu_{\mathrm{Ri}}$ appearing in Equation (18a,b) for the free energy density.

A similar model to the one presented in Equation (17) should be assumed for the dielectric permittivity of the elastomer by taking into account that the permittivity is a compliance function. However, as mentioned above, the dielectric relaxation process takes place very quickly in comparison with the viscoelastic one and, in these conditions, the dielectric relaxation process is in equilibrium, that is, the material is fully dielectrically relaxed when the viscoelastic process starts. On the other hand, it is assumed that possible conductive relaxation processes in the elastomer, which currently are proportional to $\omega^{-s},\;(0<s<1)$, are significantly active at frequencies considerably lower than the viscoelastic relaxation processes, and, for this reason, no overlapping between these two processes is expected. In Equation (17), α,β and $\tau_{1,2}$ are, respectively, the fractional-order of the ξ derivative, and the two relaxation times.

In order to obtain a better fit of the experimental results, a modified Mooney–Rivlin model has been chosen for the free energy density instead of the neo-Hookean one. Accordingly, the free energy density for the case in which viscoelastic effects are present may be written as

(18a) $$\begin{array}{c} W=\frac{\mu_{R1}}{2}\left(\lambda_{1}^{2}+\lambda_{2}^{2}+\lambda_{1}^{-2}\lambda_{2}^{-2}-3\right)+\frac{\mu_{R2}}{2}\left(\lambda_{1}^{-2}+\lambda_{2}^{-2}+\lambda_{1}^{2}\lambda_{2}^{2}-3\right)\\ +\frac{\mu_{U1}-\mu_{R1}}{2}\left(\lambda_{1}^{2}\xi_{11}^{-2}\xi_{12}^{-2}+\lambda_{2}^{2}\xi_{21}^{-2}\xi_{22}^{-2}+\lambda_{1}^{-2}\lambda_{2}^{-2}\xi_{11}^{2}\xi_{12}^{2}\xi_{21}^{2}\xi_{22}^{2}-3\right)\\ +\frac{\mu_{U2}-\mu_{R2}}{2}\left(\lambda_{1}^{-2}\xi_{11}^{2}\xi_{12}^{2}+\lambda_{2}^{-2}\xi_{21}^{2}\xi_{22}^{2}+\lambda_{1}^{2}\lambda_{2}^{2}\xi_{11}^{-2}\xi_{12}^{-2}\xi_{21}^{-2}\xi_{22}^{-2}-3\right)+\frac{{\tilde{D}}^{2}}{2\varepsilon }\lambda_{1}^{-2}\lambda_{2}^{-2} \end{array}$$

In the present approach, it is assumed that the permittivity ε does not depend on the strain. As it has been discussed in another study [40], the polarity of the acrylic VHB 4910 elastomer corresponds to a type C polymer (in the classical Stockmayer´s terminology [41]), in which the dipoles causing the main dielectric activity are in the lateral chains and, according to it, they are separated from the backbone by flexible segments, and therefore, its permittivity should be scarcely affected by stretching.

Equation (18a) is a generalisation of Equation (17) of the Ref. [4] useful for our purposes. In this equation, $\mu_{\mathrm{Ri}}$ and $\mu_{\mathrm{Ui}}$ with i=1,2 are the relaxed and unrelaxed shear moduli for the two branches of Figure 4. The first two terms on the right-hand side represent the elastic energy of the spring at the top of the scheme given in Figure 4. The third and fourth terms in this Figure correspond to the bottom branch of the before mentioned scheme. Moreover, $\xi_{\mathrm{ij}},\;i,j=1,2$ are the stretches due to the two dashpots. Two subindexes are used to describe the stretching in these two dashpots: the first one refers to each one of the two in-plane stretches, whereas the second refers to the two CPE existing in the model. The last term represents the electrostatic energy of the material, with $\tilde{D}$ being the nominal dielectric displacement. The relationship between the nominal dielectric displacement and the nominal electric field is given by $\tilde{D}={\varepsilon \lambda }_{1}^{2}\lambda_{2}^{2}Ẽ$, where ε is the dielectric permittivity, and Ẽ is the nominal electric field. In the forthcoming calculations, it is assumed that the permittivity is not dependent on the stretches. Of course, many other empirical or semiempirical models should be checked, but for the present purposes, the chosen approach suffices.

**Figure 5:**
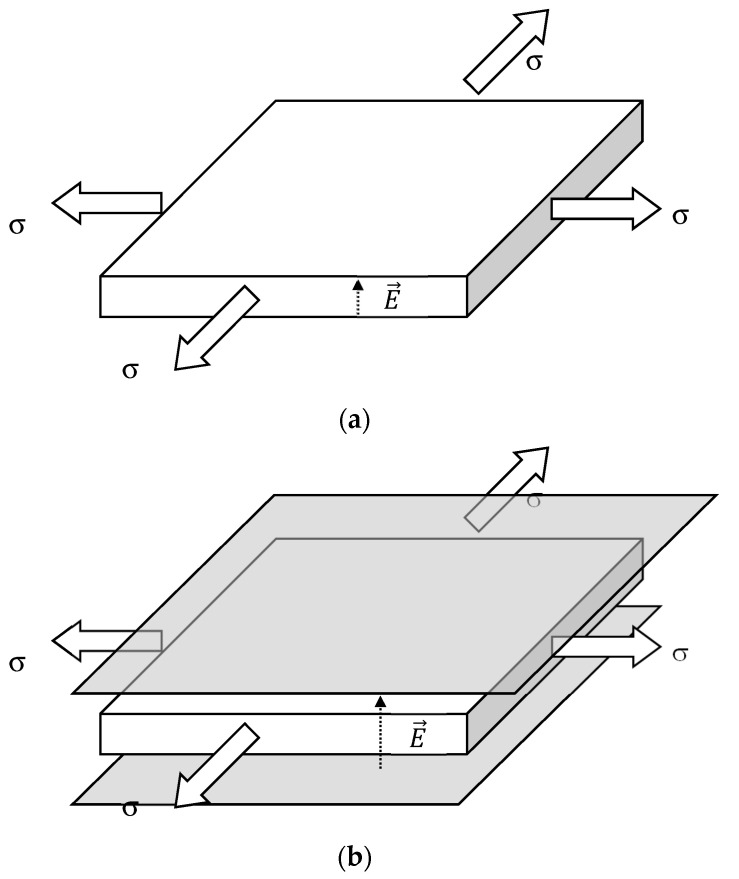
Geometric configuration of (**a**) sample with compliant electrode and (**b**) floating sample.

It is noted that Equation (18a) is valid for the case of compliant electrodes (Figure 5a), that is, for the case in which the electrodes are deposited on the two faces of the sample, by ion sputtering, for example. However, in some circumstances, for example, nematic liquid crystal elastomers [42], the constrained geometry is avoided in order to eliminate anchoring effects in the boundaries of the sample. To accomplish this purpose, a gap between the sample and the electrodes is adjusted using adequate spacers in such a way that the distance between the electrodes is maintained systematically larger than the thickness of the sample (Figure 5b). The gap should be large enough to prevent pull-in phenomena.

In that case, the Maxwell stress tensor between the sample slab and the electrodes should be taken into account, and the free energy density, taking into account the viscoelasticity, is now given by

(18b) $$\begin{array}{c} W=\frac{\mu_{R1}}{2}\left(\lambda_{1}^{2}+\lambda_{2}^{2}+\lambda_{1}^{-2}\lambda_{2}^{-2}-3\right)+\frac{\mu_{R2}}{2}\left(\lambda_{1}^{-2}+\lambda_{2}^{-2}+\lambda_{1}^{2}\lambda_{2}^{2}-3\right)\\ +\frac{\mu_{U1}-\mu_{R1}}{2}\left(\lambda_{1}^{2}\xi_{11}^{-2}\xi_{12}^{-2}+\lambda_{2}^{2}\xi_{21}^{-2}\xi_{22}^{-2}+\lambda_{1}^{-2}\lambda_{2}^{-2}\xi_{11}^{2}\xi_{12}^{2}\xi_{21}^{2}\xi_{22}^{2}-3\right)\\ +\frac{\mu_{U2}-\mu_{R2}}{2}\left(\lambda_{1}^{-2}\xi_{11}^{2}\xi_{12}^{2}+\lambda_{2}^{-2}\xi_{21}^{2}\xi_{22}^{2}+\lambda_{1}^{2}\lambda_{2}^{2}\xi_{11}^{-2}\xi_{12}^{-2}\xi_{21}^{-2}\xi_{22}^{-2}-3\right)+\left(\varepsilon^{-1}-\varepsilon_{0}^{-1}\right)\frac{{\tilde{D}}^{2}}{2}\lambda_{1}^{-2}\lambda_{2}^{-2} \end{array}$$

where $\varepsilon_{0}$ is the permittivity of the fluid where the sample is floating, which usually is the air.

Let us assume that geometry under consideration refers to a squared plate of electroviscoelastic material subjected to two pairs of forces or deformations in the principal directions. An electric field is applied perpendicular to the plate.

For equibiaxial stretches and in order to simplify calculations, it is assumed that

(19) $$\lambda_{1}=\lambda_{2}=\lambda ,{\;\xi }_{11}=\xi_{21}=\xi_{1},{\;\xi }_{12}=\xi_{22}=\xi_{2}$$

Then, the following equations for the stresses and the electric field together with two kinetic equations are found (see Appendix C).

(20a) $$\begin{array}{l} \sigma_{1}=\left(\mu_{R1}+\lambda_{2}^{2}\mu_{R2}\right)\left(\lambda_{1}-\lambda_{1}^{-3}\lambda_{2}^{-2}\right)\\ +\left[\left(\mu_{U1}-\mu_{R1}\right)+\lambda_{2}^{2}\xi_{1}^{-2}\xi_{2}^{-2}\left(\mu_{U2}-\mu_{R2}\right)\right]\left(\lambda_{1}\xi_{1}^{-2}\xi_{2}^{-2}-\lambda_{1}^{-3}\lambda_{2}^{-2}\xi_{1}^{4}\xi_{2}^{4}\right)-\varepsilon^{-1}{\tilde{D}}^{2}\lambda_{1}^{-3}\lambda_{2}^{-2}\\ \sigma_{2}=\left(\mu_{R1}+\lambda_{1}^{2}\mu_{R2}\right)\left(\lambda_{2}-\lambda_{2}^{-3}\lambda_{1}^{-2}\right)\\ +\left[\left(\mu_{U1}-\mu_{R1}\right)+\lambda_{1}^{2}\xi_{1}^{-2}\xi_{2}^{-2}\left(\mu_{U2}-\mu_{R2}\right)\right]\left(\lambda_{2}\xi_{1}^{-2}\xi_{2}^{-2}-\lambda_{2}^{-3}\lambda_{1}^{-2}\xi_{1}^{4}\xi_{2}^{4}\right)-\varepsilon^{-1}{\tilde{D}}^{2}\lambda_{2}^{-3}\lambda_{1}^{-2}\\ Ẽ=\varepsilon^{-1}{\tilde{D}\lambda }_{1}^{-2}\lambda_{2}^{-2} \end{array}$$

for Equation (17), and

(20b) $$\begin{array}{l} \sigma_{1}=\left(\mu_{R1}+\lambda_{2}^{2}\mu_{R2}\right)\left(\lambda_{1}-\lambda_{1}^{-3}\lambda_{2}^{-2}\right)\\ +\left[\left(\mu_{U1}-\mu_{R1}\right)+\lambda_{2}^{2}\xi_{1}^{-2}\xi_{2}^{-2}\left(\mu_{U2}-\mu_{R2}\right)\right]\left(\lambda_{1}\xi_{1}^{-2}\xi_{2}^{-2}-\lambda_{1}^{-3}\lambda_{2}^{-2}\xi_{1}^{4}\xi_{2}^{4}\right)-\left(\varepsilon^{-1}-\varepsilon_{0}^{-1}\right){\tilde{D}}^{2}\lambda_{1}^{-3}\lambda_{2}^{-2}\\ \sigma_{2}=\left(\mu_{R1}+\lambda_{1}^{2}\mu_{R2}\right)\left(\lambda_{2}-\lambda_{2}^{-3}\lambda_{1}^{-2}\right)\\ +\left[\left(\mu_{U1}-\mu_{R1}\right)+\lambda_{1}^{2}\xi_{1}^{-2}\xi_{2}^{-2}\left(\mu_{U2}-\mu_{R2}\right)\right]\left(\lambda_{2}\xi_{1}^{-2}\xi_{2}^{-2}-\lambda_{2}^{-3}\lambda_{1}^{-2}\xi_{1}^{4}\xi_{2}^{4}\right)-\left(\varepsilon^{-1}-\varepsilon_{0}^{-1}\right){\tilde{D}}^{2}\lambda_{2}^{-3}\lambda_{1}^{-2}\\ Ẽ=\left(\varepsilon^{-1}-\varepsilon_{0}^{-1}\right){\tilde{D}\lambda }_{1}^{-2}\lambda_{2}^{-2} \end{array}$$

for Equation (18b).

Note that the equations for Ẽ (20a) (3rd equation) and (20b) (3rd equation) are the relation between the nominal electric field and the nominal displacement, to be used later.

Then, according to the methodology outlined in Appendix C and, after the pertinent algebra, one obtains for the kinetic restriction equations,

(21a) $$\begin{array}{c} \frac{d^{\alpha }\xi_{1}}{{\mathrm{dt}}^{\alpha }}=\frac{1}{\tau_{1}^{\alpha }}\left[\left(\lambda^{2}\xi_{1}^{-3}\xi_{2}^{-2}-\lambda^{-4}\xi_{1}^{3}\xi_{2}^{4}\right)-k\left(\lambda^{-2}\xi_{1}\xi_{2}^{2}-\lambda^{4}\xi_{1}^{-5}\xi_{2}^{-4}\right)\right]\\ +\frac{1}{\tau_{2}^{\alpha }}\left[\left(\lambda^{2}\xi_{1}^{-2}\xi_{2}^{-3}-\lambda^{-4}\xi_{1}^{4}\xi_{2}^{3}\right)-k\left(\lambda^{-2}\xi_{1}^{2}\xi_{2}-\lambda^{4}\xi_{1}^{-4}\xi_{2}^{-5}\right)\right] \end{array}$$

(21b) $$\begin{array}{c} \frac{d^{\beta }\xi_{2}}{{\mathrm{dt}}^{\beta }}=\frac{1}{\tau_{1}^{\beta }}\left[\left(\lambda^{2}\xi_{1}^{-3}\xi_{2}^{-2}-\lambda^{-4}\xi_{1}^{3}\xi_{2}^{4}\right)-k\left(\lambda^{-2}\xi_{1}\xi_{2}^{2}-\lambda^{4}\xi_{1}^{-5}\xi_{2}^{-4}\right)\right]\\ +\frac{1}{\tau_{2}^{\beta }}\left[\left(\lambda^{2}\xi_{1}^{-2}\xi_{2}^{-3}-\lambda^{-4}\xi_{1}^{4}\xi_{2}^{3}\right)-k\left(\lambda^{-2}\xi_{1}^{2}\xi_{2}-\lambda^{4}\xi_{1}^{-4}\xi_{2}^{-5}\right)\right] \end{array}$$

where the initial conditions are $\xi_{1}\left(0\right)=\xi_{2}\left(0\right)=1$, and $k=\frac{\mu_{U2}-\mu_{R2}}{\mu_{U1}-\mu_{R1}}=\frac{\mu_{R2}}{\mu_{R1}}$.

It should be noted that there are many possibilities with respect to the choosing of the kinetic models to fulfil the thermodynamic requirements expressed by Equation (A14) in Appendix C. They are closely dependent on the properties of the electroelastic material under consideration.

## 5. Practical Application

For practical demonstration purposes, the acrylic elastomer VHB 4910 from 3M is the system to which the present approach is applied. Of course, other electroelastic materials should be considered.

### 5.1. Physicochemical Structure of VHB 4910

The chemical structure of VHB 4910 is formed by a two-block acrylic structure in which the length of each block and at least one of the substituted radicals in the lateral chain are commercially protected [43]. The average density of the material is 960 kg m^−3^, and the usual thickness of the tape is 1 mm.

In the following, it is considered that dielectric permittivity is not affected by stretching [40]. Despite the possible effect of the electric field on the morphological structure of the material, it is not taken into account in the present study.

### 5.2. Calorimetric Measurements

As reported in a previous paper [24], the glass transition temperature was calculated by differential scanning calorimetry (DSC) at 20 °Cmin^−1^ using a TA Instruments Q-20 calorimeter. Measurements were carried out in a dry nitrogen atmosphere in a temperature range from −80 to 140 °C. The endothermic shift of the baseline was taken as the glass temperature (Tg = −37 °C). This temperature, which is in good agreement with previous results obtained by other authors [43,44,45], is about 60 degrees below that the temperature at which the experiments are carried out. Despite that, viscoelastic activity should be expected at this temperature. In fact, a requirement in order to have a good electroelastic response in the material is to show a glass transition temperature of about 50 °C to 60 °C lower than the temperature at which the experiments are carried out (typically, the room temperature). The material under study obviously accomplishes this requirement, but this is also valid for other rubber-like materials.

### 5.3. Dynamic Mechanical Measurements

As mentioned, the room temperature at which the future calculations are carried out is more than 60 degrees above the glass transition temperature. In these circumstances, the material is too soft to obtain reliable dynamic mechanical data.

Due to the above-mentioned problem, most authors try to use indirect methods to find truly reliable data. For example, Molberg et al. [46] use, as departing point, dynamic shear data from which the dynamic tensile modulus is obtained by assuming a constant Poisson´s ratio of 0.5, corresponding to an incompressible material. The data were obtained at 23 °C in a frequency range between 0.8 mHz and 80 Hz. The obtained results do not show an indication of a maximum in the curve representing loss modulus against the frequency. Moreover, the storage modulus is fitted to an exponential function of the frequency, a very unusual procedure in dynamic mechanical experiments. On the other hand, Suo et al. [4] use, in their calculations, data from the seminal papers by Wissler et al. [19] and Planté and Dubowski [20].

In [2], Lochmatter et al. fit a visco-hyperelastic film model by using a uniaxial tensile–creep–relaxation test sequence. They also assume a single relaxation time in the main equation, but they do not report the temperature of the experiment. As pointed out by Wang et al. [7], the model parameters according to the Lochmatter approach are obtained at very low stretch rates which led to the fact that they reflect basically the static (relaxed) viscoelastic modulus. For this reason, the relaxation strength, (Kp-Ks) in the terminology used by Lochmatter et al. [2] is very low. In fact, it is a well-known fact that the relaxation strength, which depends on the number of molecular moieties suffering relaxation processes, diminishes drastically with the temperature. Wang et al. [7] follow, in many respects, the main lines of Lochmatter´s paper using an ad hoc procedure based on uniaxial tensile strain at 25 °C with larger stretch rates, but they once more only consider a single relaxation time model. Moreover, the reported value of the relaxed modulus, Ep in Wang´s terminology, is larger than the lower value observed for the dynamic viscoelastic modulus (see Table 1 of Ref. [7]), which is unlikely, despite it can be due to the fitting procedure. When the data of Wang are used to fit an equation such as Equation (14), this fact is taken into account. On the other hand, when a single relaxation time is considered, the fitting procedure is relatively easy and the representative parameters of the viscoelastic model are obtained by using the nonlinear least-squares method [7]. However, when a more complex model equation such as Equation (14) is used for the fitting procedure in order to give an account of a distribution of relaxation times, the situation is more complicated. First of all, the starting data should be the dynamic shear modulus as appears in Equation (17).

Now, the two components of the dynamic shear modulus, that is, the storage and loss modulus  μ′=Re μ*, μ ″=Im μ*, respectively, need to be obtained. This problem has been classically addressed by using the Kramers–Kronig (KK) transforms [47,48], which are a consequence of the causality principle together with the linearity of the system, which is assumed in those that follow. A good account of the use of (KK) relations for the case of dielectric relaxation has been presented by Van Turnhout [49]. According to the theory, two alternatives are possible—the first one is to relate storage and loss modulus, and the second one, chose for convenience, is to relate the phase angle $\delta \left(\omega \right)=\tan^{-1}\frac{\;\mu \prime \prime }{\mu^{\prime }}$ with the modulus |μ*| as follows [50]:

(22) $$\delta \left(\omega \right)=\frac{2\omega }{\pi }\int_{0}^{\infty }\frac{\ln\left|\mu^{*}\right|}{x^{2}-\omega^{2}}\mathrm{dx}$$

Partial integration of Equation (22) leads to

(23) $$\delta \left(\omega \right)=\frac{1}{\pi }\int_{-\infty }^{+\infty }\frac{\mathrm{dln}\left|\mu^{*}\left(x\right)\right|}{d\ln x}\ln\left|\frac{x+\omega }{x-\omega }\right|d\ln x$$

This equation has been proposed by Booij and Thoone [51]. These authors assume that the factor $\frac{\mathrm{dln}\left|\mu^{*}\left(x\right)\right|}{d\ln x}$ in (23) is nearly constant in a broad frequency band and after taking

(24) $$\int_{-\infty }^{+\infty }\ln\left|\frac{x+\omega }{x-\omega }\right|d\ln x=\frac{1}{2}\pi^{2}$$

They obtain a simplified expression for δ(ω) as

(25) $$\delta \left(\omega \right)≅\frac{\pi }{2}\frac{\mathrm{dln}\left|\mu^{*}\left(x\right)\right|}{d\ln x}$$

However, this result is only a crude approximation for the phase angle.

Consequently, it is necessary to solve by numerical integration of Equation (23). For this purpose, the first step is to fit the empirical data for the viscoelastic shear modulus |$\mu^{*}$|, obtained from Equation (21a,b) and the data of [7] to a sigmoidal-type equation as follows:

(26) $$\left|\mu^{*}\right|=\left[\mu_{R}+\frac{\mu_{U}-\mu_{R}}{1+\exp\left(-1.6113\left(x+3.7772\right)\right)}\right]\cdot 10^{6}\;\mathrm{Pa}$$

where $x=\ln\frac{\omega }{2\pi }=\ln f$.

The relaxed and unrelaxed moduli in Equation (26) are approximately given by

(27) $$\mu_{R}=1.5\cdot 10^{4}\;\mathrm{Pa};{\;\mu }_{U}=6.25\cdot 10^{4}\;\mathrm{Pa}\;$$

which are in sufficiently good agreement with the data of the Ref. [7].

It should be stressed that Equation (26) is only used to fit the data to a smooth function in order to obtain more data points for the forthcoming calculations and in any sense means a model for the viscoelastic data such as that has been previously chosen as Equation (17). In fact, Equation (26) does not yet predict the presence of two relaxation times corresponding to the high- and low-frequency side of the spectrum as appearing in the Huet model [28], represented by Equation (17).

After numerical integration of (23) using Equation (26), between the limits $x_{\max}=\ln f=-2{\;\mathrm{and}\;x}_{\min}=\ln f=-7$, the phase angle is obtained in this convenient range of frequencies from which tanδ(ω) and, subsequently, the real and imaginary parts of the dynamic shear modulus ($\;\mu^{\prime },\;\mu \prime \prime$) can be easily obtained according to

(28a) $$\mu^{\prime }=\left|\mu^{*}\right|\cos\delta \left(\omega \right)$$

(28b) $$\;\mu \prime \prime =\left|\mu^{*}\right|\sin\delta \left(\omega \right)$$

The obtained results are shown in Table 1, together with the data becoming from Ref. [7] after interpolation and using Equation (16) to obtain the shear modulus.

These data are used to fit the parameters appearing in Equation (17). The following parameters are obtained:

(29) $$\alpha =0.85,\;\beta =0.81,{\;\tau }_{1}=6.77\;s.\;,{\;\tau }_{2}=27.853s.$$

For completeness, the analytical expressions for the storage and loss modulus of the dynamic modulus from Equation (17) are given in Appendix D.

### 5.4. Dielectric Measurements

As in the case of viscoelastic relaxation data, the frequency and temperature dependence of the dielectric permittivity should be taken into account. However, due to the fact that the viscoelastic relaxation times are five or more orders of magnitude larger than their dielectric counterparts, this effect is not taken into account in the present paper.

It is noted that the methodology of calculation should be the same as in the viscoelastic case. However, due to the fact that the frequency band of the dielectric data is larger than the viscoelastic one, the fitting procedure should be presumably easier.

## 6. Effect of the Electric Field without Mechanical Forces

In order to make application of the proposed methodology of calculation, let us consider now the case where the system is subjected to an external electric force field but not to mechanical forces. Then, Equation (19) is solved together with Equations (18a) or (18b) when $\sigma_{1}=\sigma_{2}=0$ either for compliant or floating electrodes, which gives, respectively,

(30a) $$\left(\lambda^{6}-1\right)+\frac{\mu_{U1}-\mu_{R1}}{\mu_{R1}}\left(\lambda^{6}\xi_{1}^{-2}\xi_{2}^{-2}-\xi_{1}^{4}\xi_{2}^{4}\right)-\frac{\mu_{R2}}{\mu_{R1}}\left(\lambda^{2}-\lambda^{8}\right)-\frac{\mu_{U2}-\mu_{R2}}{\mu_{R1}}\left(\lambda^{2}\xi_{1}^{2}\xi_{2}^{2}-\lambda^{8}\xi_{1}^{-4}\xi_{2}^{-4}\right)-\frac{\varepsilon }{\mu_{R1}}\lambda^{8}{Ẽ}^{2}=0$$

for the case of compliant electrodes, and

(30b) $$\left(\lambda^{6}-1\right)+\frac{\mu_{U1}-\mu_{R1}}{\mu_{R1}}\left(\lambda^{6}\xi_{1}^{-2}\xi_{2}^{-2}-\xi_{1}^{4}\xi_{2}^{4}\right)-\frac{\mu_{R2}}{\mu_{R1}}\left(\lambda^{2}-\lambda^{8}\right)-\frac{\mu_{U2}-\mu_{R2}}{\mu_{R1}}\left(\lambda^{2}\xi_{1}^{2}\xi_{2}^{2}-\lambda^{8}\xi_{1}^{-4}\xi_{2}^{-4}\right)+\left(\varepsilon_{0}^{-1}-\varepsilon^{-1}\right)\frac{\varepsilon^{2}}{\mu_{R1}}\lambda^{8}{Ẽ}^{2}=0$$

for the case of floating electrodes. In Equations (30a) and (30b), Ẽ is the nominal electric field.

In the case of the modified Mooney–Rivlin model, Equations (18a) and (b) for the relaxed and unrelaxed shear moduli satisfies

(31) $$\mu =\mu_{1}+\mu_{2}$$

and using the relation

(32) $$k=\frac{\mu_{U2}}{\mu_{U1}}=\frac{\mu_{R2}}{\mu_{R1}}=0.12$$

one obtains $\mu_{U1}=5.5804,{\;\mu }_{R1}=1.3393,{\;\mu }_{U2}=0.6696,{\;\mu }_{R2}=0.1607$, which will be used in the forthcoming calculations. As a consequence of the structure of Equation (30a,b), the use of the new figures for $\mu_{U1},{\;\mu }_{R1},{\;\mu }_{U2},{\;\mu }_{R2}$ scarcely changes the obtained results with respect to the use of those of Equation (27).

The parameter k has been obtained from the data of Ref. [7] which, in time, has been taken from Figure 22 of Ref. [20] by using the classical procedure to fit the data to a Mooney–Rivlin equation (p. 103, [15]) for stretch ratios (λ) between 2 and 5.

## 7. Numerical Calculations

As the differential fractional algebraic equations (DFAE) given by (21a,b) do not have a closed solution, we approximate its solution by using a numerical method combining the fractional forward Euler method, for fractional differential equations applied to Equation (21a,b), and an iterative method to find the roots of the algebraic restriction given by Equation (30a,b). To explain the used methodology, an equation for a generic stretch ξ is considered. In this way, given a fractional initial value problem,

(33) $$\frac{d^{\alpha }\xi \left(t\right)}{{\mathrm{dt}}^{\alpha }}=f\left(t,\xi \left(t\right)\right),\;\xi \left(0\right)=\xi_{0}=1$$

By Theorem 3.1 of the Ref. [52], we can obtain the Volterra integral equation associated with the problem above, that is,

(34) $$\xi \left(t\right)={\;\xi }_{0}+(J_{0}^{\alpha }f)\left(t,\;\xi \left(t\right)\right)$$

To obtain an approximate solution, first, we consider a discretisation of the fractional integral,${\;J}_{0}^{\alpha }f$, based on an equally spaced mesh for the time variable ${\left\{t_{i}=\mathrm{ih}\right\}}_{i=0}^{N},$ with N+1 nodes defined in the interval [0,T], T>0, and h:=T/N. The numerical approximation of the fractional integral of f(t, ξ(t)) in $t_{i}$ is given by

(35) $$\begin{array}{c} (J_{0}^{\alpha }f)\left(t,\;\xi \left(t_{i+1}\right)\right)=\frac{1}{\Gamma \left(\alpha \right)}\;\int_{0}^{t_{i+1}}{\left(t_{i+1}-s\right)}^{\alpha -1}\;f\left(s,\xi \left(s\right)\right)\mathrm{ds}\;=\frac{1}{\Gamma \left(\alpha \right)}\overset{i}{\underset{j=0}{\sum }}\int_{t_{j}}^{t_{j+1}}{\left(t_{i+1}-s\right)}^{\alpha -1}\;f\left(s,\xi \left(s\right)\right)\mathrm{ds}\;\\ \approx \;\frac{1}{\Gamma \left(\alpha \right)}\overset{i}{\underset{j=0}{\sum }}f\left(t_{j},\xi \left(t_{j}\right)\right)\int_{t_{j}}^{t_{j+1}}{\left(t_{i+1}-s\right)}^{\alpha -1}\mathrm{ds}\\ =\frac{1}{\Gamma \left(\alpha +1\right)}\;\overset{i}{\underset{j=0}{\sum }}f\left(t_{j},\xi \left(t_{j}\right)\right)\left[{\left(i+1-j\right)}^{\alpha }-{\left(i-j\right)}^{\alpha }\right] \end{array}$$

Observe that, in the first step, we have split the integral on the time interval $\left[0,t_{i+1}\right]$, into i integrals on the time intervals $\left[t_{j},t_{j+1}\right]$, in the second step, we have approximated the function f(s,ξ(s)) over the whole interval $\left[t_{j},t_{j+1}\right]$ just by $f\left(t_{j},\;\xi \left(t_{j}\right)\right)$, and, finally in the last step, the remaining integral has been computed. With this approximation, we obtain the fractional forward Euler method, which is an extension of the classical Euler method as follows:

(36) $$\xi \left(t_{i+1}\right)={\;\xi }_{0}+\frac{1}{\Gamma \left(\alpha +1\right)}\overset{i}{\underset{j=0\;}{\sum }}f\left(t_{j},\xi \left(t_{j}\right)\right)\left[{\left(i+1-j\right)}^{\alpha }-{\left(i-j\right)}^{\alpha }\right]\;$$

According to our initial value problem, the fractional Euler method is given by

(37) $$\xi_{i+1}={\;\xi }_{0}+\frac{h^{\alpha }}{\Gamma \left(\alpha +1\right)}\overset{i}{\underset{j=1}{\sum }}\left[{\left(i+1-j\right)}^{\alpha \;}-{\left(i-j\right)}^{\alpha }\right]\;\left(\frac{1}{\tau^{\alpha }}\left(\lambda_{j}^{2}\xi_{j}^{-3}-\lambda_{j}^{-4}{\;\xi }_{j}^{3}\right)\right)$$

where i=0,…N−1, and $\xi_{i}$ and $\lambda_{i}$ are the approximations of ξ(t) and λ(t) in $t=t_{i}=\mathrm{ih}.$ Notice that in Equation (37), $\xi_{i+1}$ is obtained in terms of approximations constructed in previous nodes. The value of h is selected small enough to obtain a converged solution in each one of the models analysed.

These equations are valid for each one of the two ξ appearing in Equation (21a,b), and in this sense, the resulting equations are easily obtained from the former ones.

Once $\xi_{i}$ is obtained, the value of $\lambda_{i+1}$ is computed by finding the root of the equation for compliant electrodes (or their counterpart equation for floating electrodes),

(38) $$\mu_{R}\left(\lambda_{i+1}^{6}-1\right)+\left(\mu_{U}-\mu_{R}\right)\left(\lambda_{i+1}^{6}\xi_{i+1}^{-2}-\xi_{i+1}^{4}\right)-{\varepsilon \lambda }_{i+1}^{8}{Ẽ}^{2}=0$$

which is closer to $\lambda_{i}$. This root is computed numerically by using a combination of bisection, secant, and inverse quadratic interpolation methods [53].

This strategy is easily generalised when, as in our case, two parameters, $\xi_{1},\xi_{2}$, appear in the restriction Equation (30a,b).

**Figure 6:**
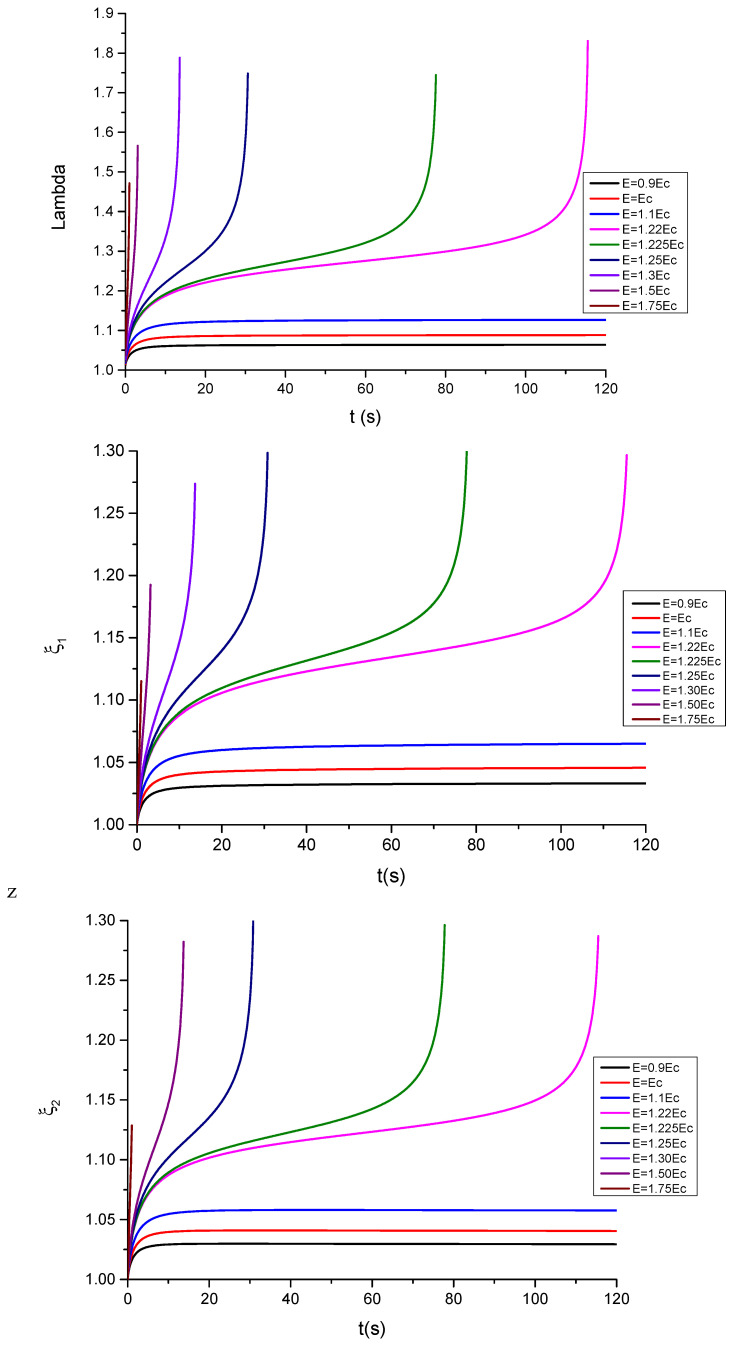
Plots showing the evolution of λ, $\xi_{1}\;\mathrm{and}\;\xi_{2}$ as a function of time for different values of the electric field E, for the case of compliant electrodes without mechanical stresses.

**Figure 7:**
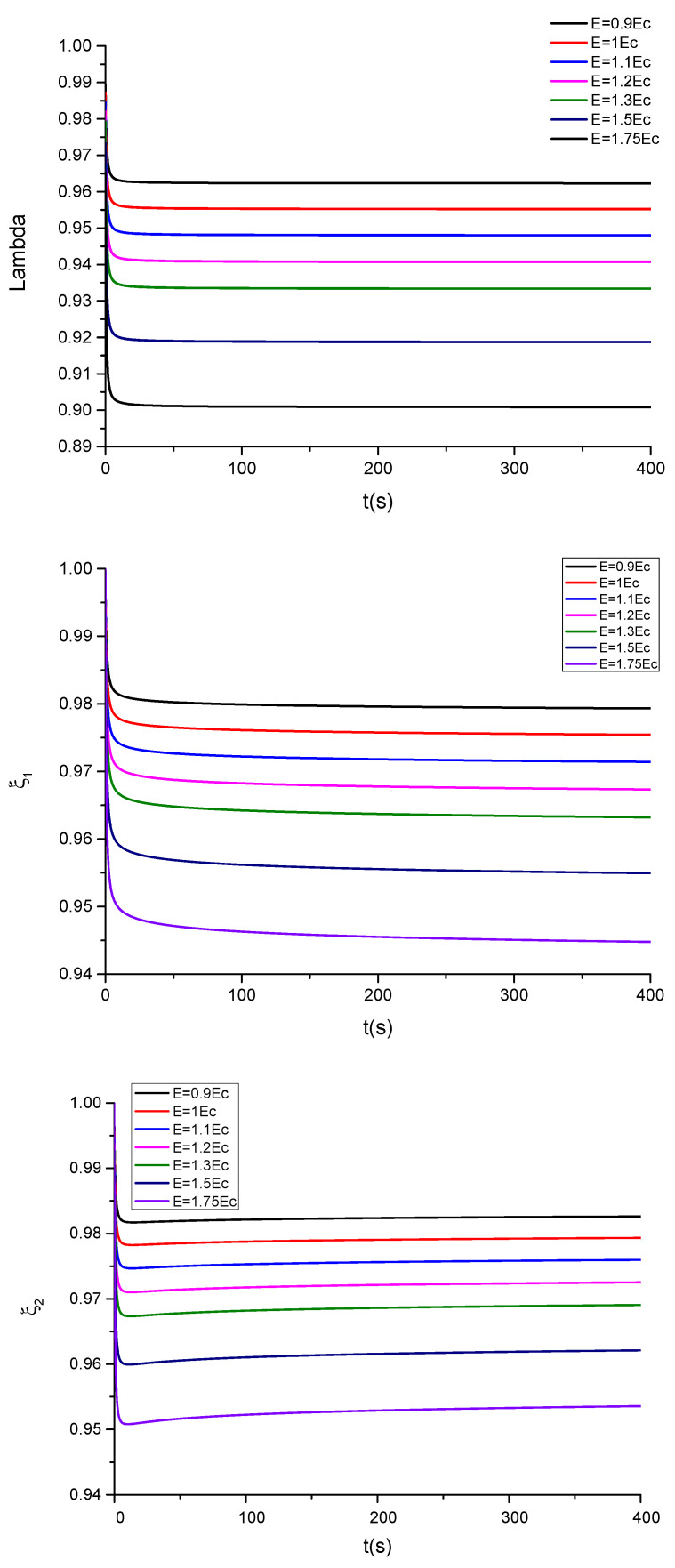
Plots showing the evolution of λ, $\xi_{1}\;\mathrm{and}\;\xi_{2}$ as a function of time for different values of the electric field E, for the case of floating electrodes without mechanical stresses.

The following parameter is used in the calculations, $\varepsilon =39.843\cdot 10^{-12}{\mathrm{Fm}}^{-1}$, being $\varepsilon_{0}=8.854\cdot 10^{-12}{\mathrm{Fm}}^{-1}$ the permittivity of the evacuated space. The critical electric field is given by ${Ẽ}_{c}=0.6203\sqrt{\frac{\mu_{R_{1}}}{\varepsilon }}$ (see Appendix E). The corresponding results for the time evolution of $\lambda ,\xi_{1},\xi_{2}$ are shown in Figure 6 and Figure 7 for, respectively, compliant and floating electrodes and several electric fields.

The calculations have also been carried out for the case of a neo-Hookean model with a viscoelastic response in compliant mode governed by a single relation time (α=1,τ=5.27 s), together with the following parameters: $\mu_{R}=1.5\times 10^{4}\mathrm{Pa},{\;\mu }_{U}=6.25\times 10^{4}\mathrm{Pa}$ as in the Equation (27) and ${Ẽ}_{c}=0.6873\sqrt{\frac{\mu_{R1}}{\varepsilon }}$ as indicated in Appendix E and in agreement with Ref. [44]. The corresponding equations are

(39) $$\left(\lambda^{6}-1\right)+\frac{\mu_{U}-\mu_{R}}{\mu_{R}}\left(\lambda^{6}\xi^{-2}-\xi^{4}\right)-\frac{\varepsilon }{\mu_{R}}\lambda^{8}{Ẽ}^{2}=0$$

with the corresponding kinetic equation given by

(40) $$\frac{d\xi }{\mathrm{dt}}=\frac{1}{\tau }\left[\lambda^{2}\xi^{-3}-\lambda^{-4}\xi^{3}\right]$$

**Figure 8:**
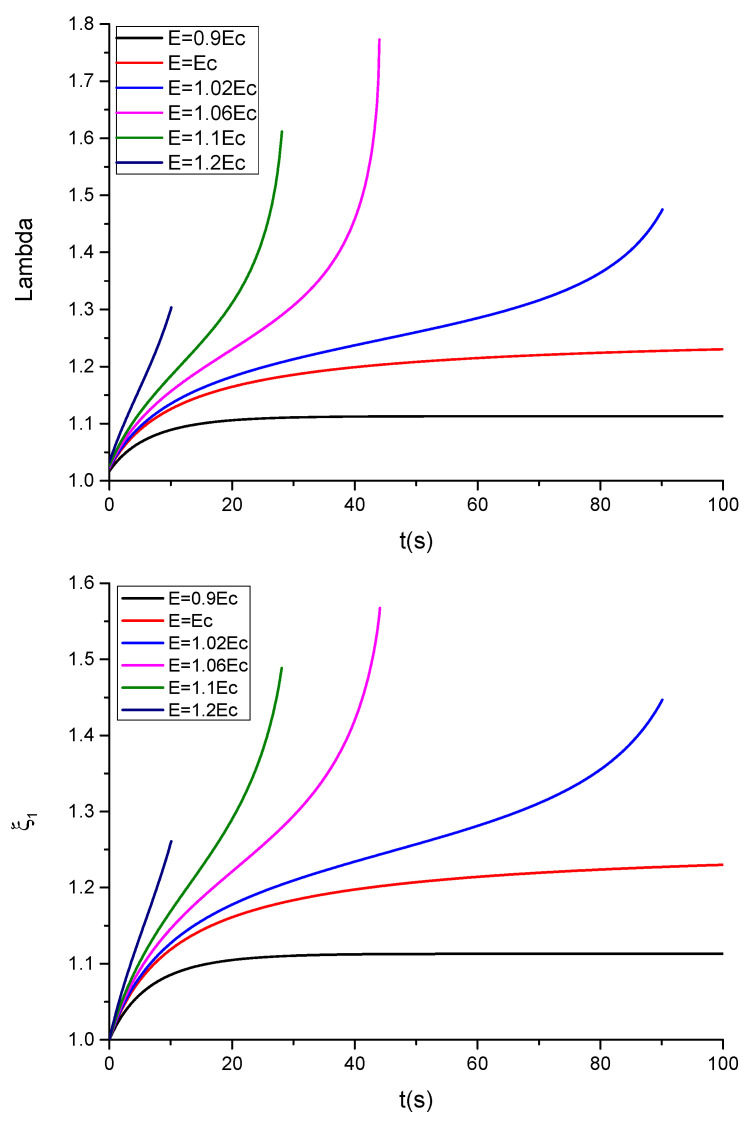
Plots showing the evolution of λ and ξ, as a function of time for different values of the electric field E, for the case of a neo-Hookean material with compliant electrodes without mechanical stresses.

The results are shown in Figure 8.

For purpose of comparison, the results obtained by Suo et al. for a single relaxation time have been recovered by using our calculation program and taking into account the following parameters taken from Ref. [4] $\mu_{U}=8.78\times 10^{4}\;\mathrm{Pa},{\;\mu }_{R}=2.28\times 10^{4}\;\mathrm{Pa},\;\alpha =1,\;\tau =200\;s.$, together with a value of ε=4.7 for the relative permittivity, as reported by Wissler and Mazza in Ref. [16]. The agreement is excellent.

## 8. Stability and Bifurcation Analysis

In the linear theory of elasticity, solutions of traction boundary problems are unique, and the stress fields are also unique. This is a consequence of the linearity of the constitutive equations relating stress and strain. However, under finite deformations, the stress–strain relationships for rubbers are nonlinear, and unlike the linear theory, the uniqueness of the solutions is, a priori, not warranted. Concomitantly, the appearance of instabilities and possible bifurcation phenomena are expected. For this reason, stability analysis in order to check the possibility of the appearance of bifurcations is pertinent because nonlinear elasticity is exhibited by these electroelastic materials.

An analysis of the instability and possible bifurcation phenomena requires using Equation (18a) as starting point because in the case of the neo-Hookean model no instability and subsequent bifurcation, as expected, are predicted. The classical procedure outlined in [54,55] and based on the Hessian approach is followed here. Let us consider, as above, a slab whose thickness in the third direction is small, in comparison with the lateral dimensions subjected to two pairs of dead loads in these two directions. Moreover, a homogeneous electric field is applied in the third direction. Since the deformation and the field are basically homogeneous, the field equations are automatically fulfilled. For the case of equal dead loads on the four lateral surfaces, one has for each of Equation (18a)

(41) $$\frac{\partial W}{\partial \lambda_{1}}=\frac{\partial W}{\partial \lambda_{2}}$$

After the pertinent algebraic calculations, the subsequent equation which can be factorised to give a symmetric solution defined by

(42) $$\lambda_{1}=\lambda_{2}$$

that represents the bisector of the first quadrant in a $\lambda_{1}\;\mathrm{vs}.{\;\lambda }_{2}$ plot. Moreover, a nonsymmetric solution is found, which intersects with the symmetric solution after taking into account (19) for the values of λ solving the following equations for, respectively, compliant and floating electrodes

(43a) $$\left(\lambda^{6}+1\right)+\frac{\mu_{U1}-\mu_{R1}}{\mu_{R1}}\left(\lambda^{6}\xi_{1}^{-2}\xi_{2}^{-2}+\xi_{1}^{4}\xi_{2}^{4}\right)+\frac{\mu_{R2}}{\mu_{R1}}\left(3\lambda^{2}-\lambda^{8}\right)+\frac{\mu_{U2}-\mu_{R2}}{\mu_{R1}}\left(3\lambda^{2}\xi_{1}^{2}\xi_{2}^{2}-\lambda^{8}\xi_{1}^{-4}\xi_{2}^{-4}\right)+{\varepsilon \mu }_{R_{1}}^{-1}\lambda^{8}{Ẽ}^{2}=0$$

(43b) $$\left(\lambda^{6}+1\right)+\frac{\mu_{U1}-\mu_{R1}}{\mu_{R1}}\left(\lambda^{6}\xi_{1}^{-2}\xi_{2}^{-2}+\xi_{1}^{4}\xi_{2}^{4}\right)+\frac{\mu_{R2}}{\mu_{R1}}\left(3\lambda^{2}-\lambda^{8}\right)+\frac{\mu_{U2}-\mu_{R2}}{\mu_{R1}}\left(3\lambda^{2}\xi_{1}^{2}\xi_{2}^{2}-\lambda^{8}\xi_{1}^{-4}\xi_{2}^{-4}\right)-\left(\varepsilon_{0}^{-1}-\varepsilon^{-1}\right)\varepsilon^{2}\mu_{R_{1}}^{-1}\lambda^{8}{Ẽ}^{2}=0$$

**Figure 9:**
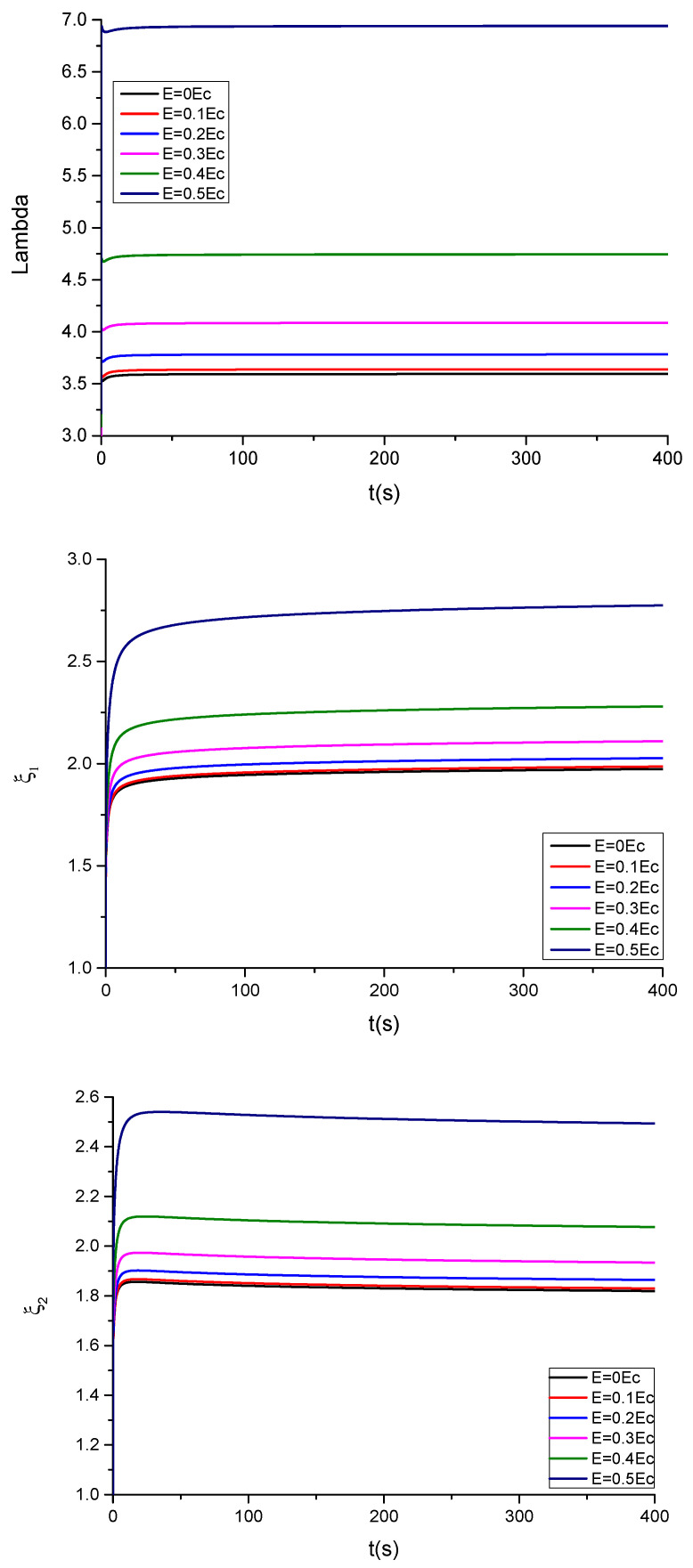
Plot of the bifurcation values of λ, $\xi_{1},{\;\xi }_{2}$ as a function of time for different values of the electric field E, for the case of a material with an energy function given by Equation (18a) (compliant electrodes).

**Figure 10:**
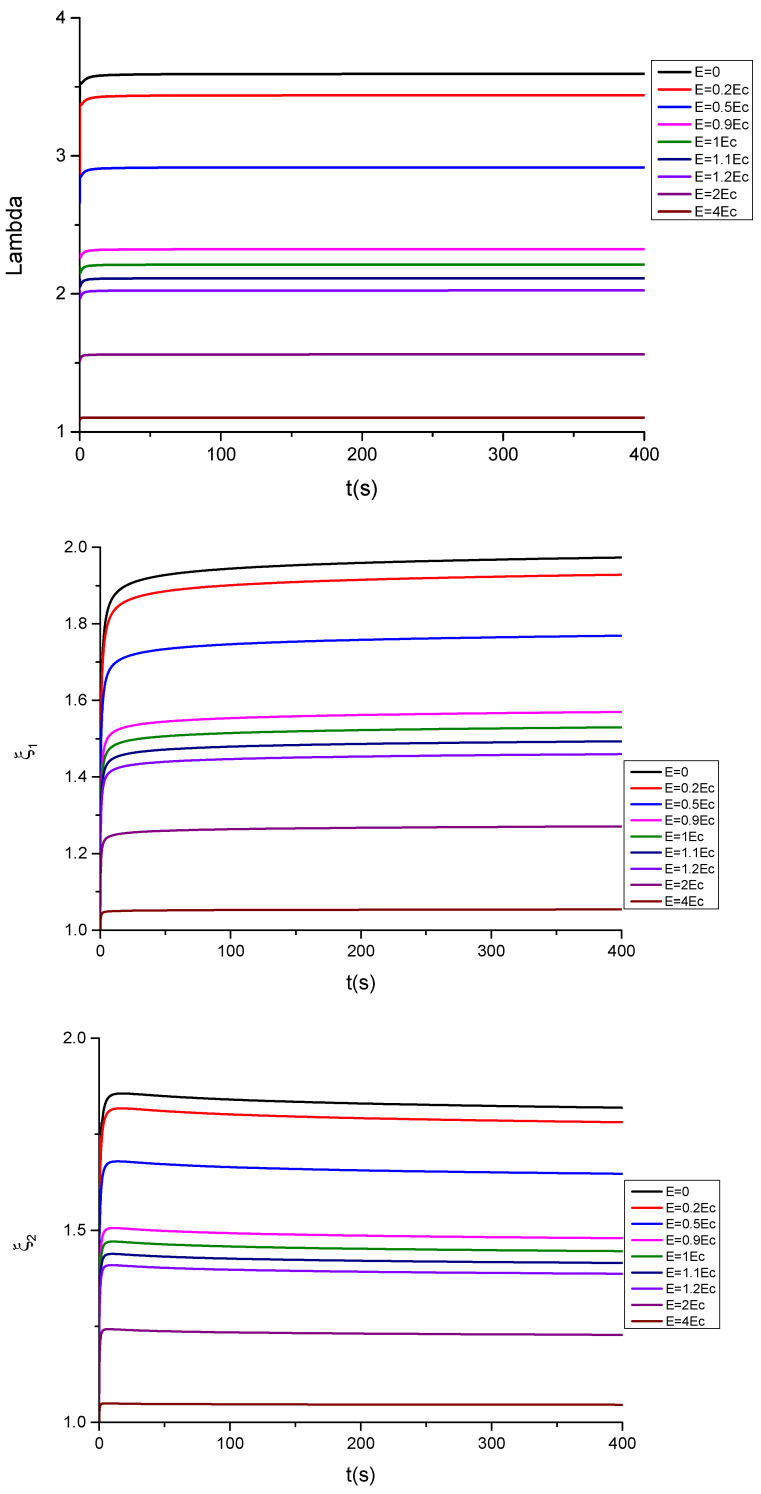
Plot of the bifurcation values of λ, $\xi_{1},{\;\xi }_{2}$ as a function of time for different values of the electric field E, for the case of a material with an energy function given by Equation (18b) (floating electrodes).

The time-dependent parameters $\lambda ,\xi_{1},\xi_{2}$ at which the bifurcation occurs are obtained by solving Equation (43a,b), together with Equation (21b). The parameters for the Equation (43a,b) used in the corresponding numerical calculations are the same as the used in the previous calculations. Figure 9 and Figure 10, respectively, show the effect of the electric field on the time evolution of the bifurcation stretch λ and $\xi_{1},\xi_{2}$ for compliant and floating electrodes because we are dealing with a time-dependent bifurcation. Of course, other bifurcation modes and more complex bifurcation maps should be obtained by using, for example, incremental methods (see, for example, Ref. [56]), but for the present purposes, the approach used here is sufficient.

## 9. Discussion

The plot of the stretching λ and of the parameters $\xi_{1},\xi_{2}$ of the dielectric elastomer under study for several applied electric fields in the compliant configuration is, respectively, shown in Figure 6 and Figure 7. In the case of compliant electrodes (Figure 6), starting from t = 0, the material relaxes with time, and subsequently, the stretch increases. For $Ẽ<{Ẽ}_{c}$, the observed parameters tend to a new equilibrium state, but for $Ẽ\geq {Ẽ}_{c}$, the elastomer becomes unstable at progressively higher electric fields. These results are in qualitative agreement with those observed in [4]. However, as can be seen in Figure 7, in the case of floating electrodes, the values of $\lambda ,{\;\xi }_{1},\xi_{2}$ are lower than the unity decreasing with the time until an equilibrium value. This is due to the fact that the material sample is in compression under the effect of progressively higher electric fields.

In the case of a neo-Hookean material governed by a single relaxation time corresponding to Figure 8, the instability appears at lower times, as previously observed [4]. This fact suggests that the use of a more realistic model than the Mooney–Rivlin model is a more realistic strategy to achieve valuable results in the study of these materials.

The bifurcation analysis, which is shown in Figure 9 and Figure 10, indicates that the values of $\lambda ,{\;\xi }_{1},\xi_{2}$ at the bifurcation increase with the time for the case of compliant, as well as for the floating electrodes. It should be noted that for $\xi_{1}=\xi_{2}=0$ and in absence of electric field, a value of λ=2.897 is obtained with $\frac{\mu_{R}}{\mu_{U}}=0.12$ from the following bifurcation equation:

(44) $$\left(\lambda^{6}+1\right)+0.12\left(3\lambda^{2}-\lambda^{8}\right)=0$$

in agreement with the classical results. However, this value increases (diminishes) with the electric field for the case of compliant (floating) electrodes, in qualitative agreement with the results obtained in Ref. [54].

## 10. Conclusions

In this paper, as usual, an empirical model for the free energy density for a visco-hyperelastic material, VHB 4910, has been used as an example. In the present approach, no modelling is carried out on the possible electrical relaxation or conduction processes, because, as mentioned in the Introduction Section, when the viscoelastic relaxation processes take place, their electrical counterparts have occurred several decades before. Moreover, the treatment of the viscoelastic effects on the response of electroelastic materials, as outlined by Zhao and Suo [54], is modified in order to consider (a) the Mooney–Rivlin model instead of the neo-Hookean one, (b) a distribution of relaxation times instead of a single relaxation time, and (c) the asymmetric character [27] of the observed viscoelastic relaxations instead of a symmetric (CC) arc [26]. For this purpose, fractional derivatives are used in order to construct the respective constitutive equations, as explained in Section 3. The results obtained in the present paper suggest that the use of fractional derivatives for modelling the viscoelastic behaviour of electroelastic materials can give an account of the distribution of dielectric relaxation times. This seems to be a more realistic approach than those previously used to calculate the time evolution of the stretch λ and the viscoelastic parameters $\xi_{1},\xi_{2}$ of these materials on the basis of a single relaxation time. Moreover, from the experimental point of view, the cases of compliant as well as floating electrodes are analysed. Both cases give very different results.

In order to compare our results with the former studies, the calculations are also carried out for the case of a neo-Hookean model with a viscoelastic model governed by a single relaxation time taking into account the parameters as in [4], and the agreement is very good.

On the other hand, the classical bifurcation analysis carried out for compliant as well as floating samples show results that are in agreement with those previously obtained for electroelastic, nonviscoelastic materials (Ref. [54]). It is to be stressed that the results obtained should potentially be useful in the design of electroelastic sensors and transducers as well as biomedical applications [57,58], prostheses, and robots, for example, in the case of cylindrical geometry for aortic prosthesis [59]. This is one of the main highlights of the present study, that is, to prevent the appearance of unexpected anomalous electroelastic responses.

## Figures and Tables

**Table 1:** Calculated values for viscoelastic parameters as a function of the frequency.

	Values from [7]			This Paper		
ln f	tan δ	μ′ × 10^−4^ (Pa)	μ′′ × 10^−4^ (Pa)	tan δ	μ′ × 10^-4^ (Pa)	μ′′ × 10^−4^ (Pa)
−7				0.11	1.52	1.13
−6.5				0.18	1.54	0.28
−6	0.25	1.60	0.40	0.27	1.57	0.42
−5.5	0.38	1.70	0.65	0.39	1.66	0.65
−5	0.53	1.82	0.96	0.54	1.83	0.99
−4.5	0.68	2.156	1.46	0.68	2.17	1.48
−4	0.70	2.78	1.95	0.70	2.63	1.98
−3.5	0.54	3.75	2,03	0.55	3.65	2.12
−3	0.39	4.80	1.87	0.40	4.79	1.92
−2.5	0.28	5.51	1.54	0.28	5.51	1.54
−2	0.19	5.91	1.12	0.18	5.90	1.06
−1.5				0.10	6.10	0.61

## Data Availability

The data presented in this study are available on request from the corresponding author.

## Appendix A

A constant phase element (CPE) is defined as a complex electrical impedance of the type [13]

(A1) $$Z_{\mathrm{CPE}}^{*}=\frac{\tau_{0}}{\varepsilon_{0}\left(\varepsilon_{s}-\varepsilon_{\infty }\right)}{\left({j\omega \tau }_{0}\right)}^{-\alpha }$$

where $\tau_{0}$ is the dielectric relaxation time, $\varepsilon_{0}$ is the permittivity of the evacuated space, $\varepsilon_{s},{\;\varepsilon }_{\infty }$ are, respectively, the relaxed and unrelaxed permittivity, j is the imaginary unity, ω the angular frequency, and 0≤α<1.

The corresponding electrical admittance is given by

(A2) $$Y_{\mathrm{CPE}}^{*}=\frac{{\left({j\omega \tau }_{0}\right)}^{\alpha }}{R}$$

where $R=\frac{\tau_{0}}{\varepsilon_{0}\left(\varepsilon_{s}-\varepsilon_{\infty }\right)}$.

Then, the electrical admittance of the model represented in Figure 3 is given by

(A3) $$Y^{*}={j\omega C}_{1}+\frac{{j\omega C}_{2}}{\left[1+C_{2}A{\left(j\omega \right)}^{1-\alpha }\right]}$$

From which the Cole–Cole equation is easily obtained by putting $C_{2}A=\tau^{1-\alpha },{\;C}_{1}=\varepsilon_{0}\varepsilon_{\infty },{\;C}_{2}=\varepsilon_{0}\left(\varepsilon_{s}-\varepsilon_{\infty }\right),{\;C}_{1}=\varepsilon_{\infty }C_{0}$, dividing both sides of Equation (A5) by ${j\omega C}_{0}$ and writing $\frac{Y^{*}}{{j\omega C}_{0}}=\varepsilon^{*}$, where $\varepsilon^{*}$ is the complex dielectric permittivity.

The mechanical counterpart of Equations (A2) and (A3) are written in a similar form (see Equations (6) and (7) of the paper). These definitions are consistent with the Caputo derivative as defined in Equation (11) and explain why the use of a power–law kernel is appropriate in the main body of the paper.

## Appendix B

According to Figure 2, the total stress in the system is

(A4) $$\sigma =\sigma_{1}+\sigma_{2}$$

where $\sigma_{1}=\mu_{R}\varepsilon$ and $\sigma_{2}$ are, respectively, the stress in the upper spring and the Maxwell element at the bottom of Figure 2.

The total deformation in the Maxwell element will be

(A5) $$\varepsilon =\varepsilon_{1}+\varepsilon_{2}$$

where $\varepsilon_{1}$ and $\varepsilon_{2}$ are, respectively, the deformations in the spring and in the dashpot. Obviously, one has

(A6) $$\frac{d^{\alpha }\varepsilon }{{\mathrm{dt}}^{\alpha }}=\frac{d^{\alpha }\varepsilon_{1}}{{\mathrm{dt}}^{\alpha }}+\frac{d^{\alpha }\varepsilon_{2}}{{\mathrm{dt}}^{\alpha }}$$

Taking into account Equation (7), and the stress in the spring in the Maxwell element given by

(A7) $$\sigma_{2}=\left(\mu_{U}-\mu_{R}\right)\varepsilon_{1}$$

and substitution of $\varepsilon_{1}$ and $\varepsilon_{2}$ in the right-hand side of Equation (A6) in terms of the common stress for the spring and the dashpot of the Maxwell element, $\sigma_{2}$, the following fractional differential equation is obtained:

(A8) $$\frac{d^{\alpha }\varepsilon }{{\mathrm{dt}}^{\alpha }}=\frac{1}{\mu_{U}-\mu_{R}}\frac{d^{\alpha }\sigma_{2}}{{\mathrm{dt}}^{\alpha }}+\frac{\sigma_{2}}{\eta }$$

After substitution of $\sigma_{2}=\sigma -\mu_{R}\varepsilon$ in Equation (A7) and some rearrangements, one obtains Equation (9). Now, taking the Laplace transform of Equation (9) for the case of a strain input $\epsilon =\epsilon_{0}H\left(t\right)$, as above, one recovers Equation (7).

In the last calculation, the following result has been used:

(A9) $$L\left(\frac{d^{\alpha }f\left(t\right)}{{\mathrm{dt}}^{\alpha }};s\right)=s^{\alpha }F\left[f\left(t\right);s\right]-\overset{\alpha -1}{\underset{k=0}{\sum }}s^{\alpha -k-1}f^{\left(k\right)}\left(0\right)=s^{\alpha }F\left[f\left(t\right);s\right]\overset{\alpha -1}{\underset{k=0}{\sum }}s^{k}f^{\left(n-k-1\right)}\left(0\right)$$

where L means the Laplace transform [19] (p. 15).

## Appendix C

Starting from the free energy density for incompressible elastomer $\left(\lambda_{1}\lambda_{2}\lambda_{3}=1\right)$ given by

(A10) $$W=\hat{W}\left(\lambda_{1},\lambda_{2},\tilde{D},{\;\xi }_{\mathrm{ij}},\ldots \right),\;i,j=1,2$$

the availability function A should be such that

(A11) $$\delta W-\sigma_{1}{\delta \lambda }_{1}-\sigma_{2}{\delta \lambda }_{2}-Ẽ\;\delta \tilde{D}+\underset{\mathrm{ij}}{\sum }\frac{\partial \tilde{W}}{\partial \xi_{\mathrm{ij}}}{\delta \xi }_{\mathrm{ij}}\leq 0$$

which, for this specific case, is the version of the reduced dissipation inequality and where $\sigma_{1}=\frac{\partial \tilde{W}}{\partial \lambda_{1}},{\;\sigma }_{2}=\frac{\partial \tilde{W}}{\partial \lambda_{2}},Ẽ=\frac{\partial \tilde{W}}{\partial \tilde{D}}$ are the nominal principal stresses, $Ẽ,\tilde{D}$ the nominal electric field and displacement, and the internal variables $\xi_{\mathrm{ij}}$ are identified with the stretches in the dashpots appearing in the mechanical models.

In equilibrium, and according to Equations (15) and (16), with the restrictions given by (17), the inequality given by (A11) leads to

(A12) $$\sum_{i}\frac{\partial \tilde{W}}{\partial \xi_{i}}{\delta \xi }_{i}\leq 0$$

Then, the energy is dissipated at the positive rate

(A13) $$-\underset{i}{\sum }\frac{\partial \tilde{W}}{\partial \xi_{i}}\frac{{d\xi }_{i}}{\mathrm{dt}}=\underset{i}{\sum }M_{\mathrm{ij}}\frac{\partial \tilde{W}}{\partial \xi_{i}}\frac{\partial \tilde{W}}{\partial \xi_{j}}\geq 0$$

where $M_{\mathrm{ij}}$, i,j=1,2 is a positive definite matrix which, in principle, may be dependent on the variables appearing in Equations (15b) and (16).

For a fractional order for the kinetic model, one conveniently adopts the following equation:

(A14) $$\frac{d^{k}\xi_{i}}{{\mathrm{dt}}^{k}}=-\frac{1}{2}\sum M_{\mathrm{ij}}\frac{\partial W}{\partial \xi_{j}},\;k=\alpha ,\beta ,i,j=1,2$$

where each $M_{\mathrm{ij}}$ is the inverse of a pseudo-viscosity, as defined in (8). Then, Equation (A14) may be written

(A15) $$\left(\begin{array}{c} \frac{d^{\alpha }\xi_{1}}{{\mathrm{dt}}^{\alpha }}\\ \frac{d^{\beta }\xi_{2}}{{\mathrm{dt}}^{\beta }} \end{array}\right)=-\frac{1}{2}\left(\begin{array}{cc} M_{11} & M_{12}\\ M_{21} & M_{22} \end{array}\right)\left(\begin{array}{c} \frac{\partial W}{\partial \xi_{1}}\\ \frac{\partial W}{\partial \xi_{2}} \end{array}\right)$$

from which Equations(18) is obtained where

(A16) $$\tau_{1}^{\alpha }={\left[M_{11}\left(\mu_{U_{1}}-\mu_{R_{1}}\right)\right]}^{-1},{\;\tau }_{2}^{\alpha }={\left[M_{12}\left(\mu_{U_{1}}-\mu_{R_{1}}\right)\right]}^{-1},{\;\tau }_{1}^{\beta }={\left[M_{21}\left(\mu_{U_{1}}-\mu_{R_{1}}\right)\right]}^{-1},{\;\tau }_{2}^{\beta }={\left[M_{22}\left(\mu_{U_{1}}-\mu_{R_{1}}\right)\right]}^{-1}$$

## Appendix D

After the pertinent algebra, the real and imaginary parts of the dynamic tensile modulus are obtained as follows:

(A17) $$\begin{array}{c} \;\mu^{\prime }=\mu_{R}+\left(\mu_{U}-\mu_{R}\right)\cdot \\ \left[\frac{1+\gamma {\left({\omega \tau }_{0}\right)}^{-k}\cos\frac{\pi }{2}k+{\left({\omega \tau }_{0}\right)}^{-h}\cos\frac{\pi }{2}h}{1+\gamma^{2}{\left({\omega \tau }_{0}\right)}^{-2k}+{\left({\omega \tau }_{0}\right)}^{-2h}+2\left(\gamma {\left({\omega \tau }_{0}\right)}^{-k}\cos\frac{\pi }{2}k+{\left({\omega \tau }_{0}\right)}^{-h}\cos\frac{\pi }{2}h+\gamma {\left({\omega \tau }_{0}\right)}^{-\left(k+h\right)}\cos\frac{\pi }{2}\left(h-k\right)\right)}\right] \end{array}$$

(A18) $$\begin{array}{c} \;\mu \;\prime \prime =\left(\mu_{U}-\mu_{R}\right)\cdot \\ \left[\frac{\gamma {\left({\omega \tau }_{0}\right)}^{-k}\sin\frac{\pi }{2}k+{\left({\omega \tau }_{0}\right)}^{-h}\sin\frac{\pi }{2}h}{1+\gamma^{2}{\left({\omega \tau }_{0}\right)}^{-2k}+{\left({\omega \tau }_{0}\right)}^{-2h}+2\left(\gamma {\left({\omega \tau }_{0}\right)}^{-k}\cos\frac{\pi }{2}k+{\left({\omega \tau }_{0}\right)}^{-h}\cos\frac{\pi }{2}h+\gamma {\left({\omega \tau }_{0}\right)}^{-\left(k+h\right)}\cos\frac{\pi }{2}\left(h-k\right)\right)}\right] \end{array}$$

The corresponding expressions for the dielectric permittivity can be found in Ref. [33].

## Appendix E

If no viscoelastic effects occur in the material (t=0), and the mechanical forces are absent $\left(\sigma_{1}=\sigma_{2}=0\right)$, the following equations are obtained from Equations (30a) and (30b):

(A19) $$\sqrt{\left(\lambda^{-2}-\lambda^{-8}\right)+\frac{\mu_{R2}}{\mu_{R1}}\left(\lambda^{-6}-1\right)}=\frac{Ẽ}{\sqrt{\frac{\mu_{R1}}{\varepsilon }}}$$

(A20) $$\sqrt{\left(\lambda^{-2}-\lambda^{-8}\right)+\frac{\mu_{R2}}{\mu_{R1}}\left(\lambda^{-6}-1\right)}={\left[\left(\varepsilon_{0}^{-1}-\varepsilon^{-1}\right)\frac{\epsilon^{2}}{\mu_{R1}}\right]}^{1/2}Ẽ$$

Maximising Ẽ with respect to λ, a critical stretch $\lambda^{c}=1.2296$ is obtained, when $\frac{\mu_{R2}}{\mu_{R1}}=0.12$. In the case of compliant electrodes, for comparison purposes, the critical nominal electric field is ${Ẽ}_{c}=0.6203\sqrt{\frac{\mu_{R1}}{\varepsilon }}$ which, for the stipulated values of $\mu_{R1}$ and ε, give ${Ẽ}_{c}=1.2036\times 10^{7}V/m$. In the case of a Neo-Hookean material, $\mu_{R2}=0$, and a value for the critical field of $\frac{\tilde{E_{c}}}{\sqrt{\frac{\mu_{U}}{\varepsilon }}}=0.6873$ has been obtained. Below this value of the electric field, electromechanical instability will not occur, as stated in [60].
